# Could Infectious Agents Play a Role in the Onset of Age-related Macular Degeneration? A Scoping Review

**DOI:** 10.1016/j.xops.2024.100668

**Published:** 2024-11-30

**Authors:** Petra P. Larsen, Virginie Dinet, Cécile Delcourt, Catherine Helmer, Morgane Linard

**Affiliations:** 1 University of Bordeaux, INSERM, BPH, U1219, Bordeaux, France; 2 INSERM, Biologie des Maladies Cardiovasculaires, U1034, University of Bordeaux, Pessac, France

**Keywords:** Age-related macular degeneration, Bacteria, Fungi, Risk factors, Viruses

## Abstract

**Topic:**

This scoping review aims to summarize the current state of knowledge on the potential involvement of infections in age-related macular degeneration (AMD).

**Clinical relevance:**

Age-related macular degeneration is a multifactorial disease and the leading cause of vision loss among older adults in developed countries. Clarifying whether certain infections participate in its onset or progression seems essential, given the potential implications for treatment and prevention.

**Methods:**

Using the PubMed database, we searched for articles in English, published until June 1, 2023, whose title and/or abstract contained terms related to AMD and infections. All types of study design, infectious agents, AMD diagnostic methods, and AMD stages were considered. Articles dealing with the oral and gut microbiota were not included but we provide a brief summary of high-quality literature reviews recently published on the subject.

**Results:**

Two investigators independently screened the 868 articles obtained by our algorithm and the reference lists of selected studies. In total, 40 articles were included, among which 30 on human data, 9 animal studies, 6 in vitro experiments, and 1 hypothesis paper (sometimes with several data types in the same article). Of these, 27 studies were published after 2010, highlighting a growing interest in recent years. A wide range of infectious agents has been investigated, including various microbiota (nasal, pharyngeal), 8 bacteria, 6 viral species, and 1 yeast. Among them, most have been investigated anecdotally. Only *Chlamydia pneumoniae*, *Cytomegalovirus*, and hepatitis B virus received more attention with 17, 6, and 4 studies, respectively. Numerous potential pathophysiological mechanisms have been discussed, including (1) an indirect role of infectious agents (i.e. a role of infections located distant from the eye, mainly through their interactions with the immune system) and (2) a direct role of some infectious agents implying potential infection of various cells types within AMD-related tissues.

**Conclusions:**

Overall, this review highlights the diversity of possible interactions between infectious agents and AMD and suggests avenues of research to enrich the data currently available, which provide an insufficient level of evidence to conclude whether or not infectious agents are involved in this pathology.

**Financial Disclosure(s):**

Proprietary or commercial disclosure may be found in the Footnotes and Disclosures at the end of this article.

Age-related macular degeneration (AMD) is a progressive retinal disease^1^ whose worldwide prevalence has been estimated at 8.7% in pooled global data of people between 45 and 85 years, which is expected to affect 288 million people by 2040.^2^ Age-related macular degeneration is clinically classified into 3 stages according to the severity of fundus lesions.^3^ Early AMD rarely causes visual impairment and is described by medium-sized drusen, clinically visible deposits underneath the retinal pigment epithelium (RPE). These deposits are made of lipids (esterified and unesterified cholesterol), at least 129 different proteins (e.g. β-amyloid, apolipoproteins, complement regulation factors), metal ions (zinc, iron), RPE cell debris, and immune cells.^1^ Further disease progression to intermediate AMD is associated with larger drusen and pigmentary abnormalities of the macula. Advanced AMD includes geographic atrophy (GA) and neovascular AMD: GA is characterized by gradually expanding retinal thinning due to loss of photoreceptors, RPE cells, and choroidal capillaries.^1^ Neovascular AMD corresponds to an invasion of new choroidal blood vessels (choroidal neovascularization [CNV]) into the subretinal and/or sub-RPE space that can lead to sight-threatening exudation of fluid and/or blood. Both GA and neovascular AMD result in substantial visual decline in most patients and can also coexist in 1 eye. Therapeutic options remain limited. For the treatment of neovascular AMD, intravitreal therapy using anti-VEGF has proven its ability to reduce the incidence of AMD-related legal blindness. However, life-long injections are often required, which cannot always prevent a slow deterioration of vision.^4^^,^^5^ For the treatment of GA, pegcetacoplan, a complement C3 inhibitor, was approved in 2023 in the United States, following the observation of reduced GA growth compared with sham treatment.^6^^,^^7^ However, to date, no effective therapies for early or intermediate AMD exist and none of the approved therapies for advanced AMD are curative.

The pathophysiology of AMD is complex and the underlying mechanisms are not yet fully understood. Mitochondrial dysfunction together with oxidative stress and defects in autophagy have been described to contribute to AMD.^8^ A significant inflammatory component involving the complement system and various types of immune cells (microglia, macrophages, dendritic cells) have been also implicated in AMD.^9^ Large epidemiological studies have also revealed several risk factors of AMD including age, genetics,^10^ and some environmental factors.^1^^,^^11^ The genetic risk of AMD is considered to be linked to multiple genetic loci of small to modest effect contributing to the disease. However, 2 loci have been consistently strongly linked to advanced AMD in genome-wide association studies: Complement factor H (CFH) gene at the 1q31 susceptibility locus, where the C allele of CFH Y402H is recognized as a risk factor for AMD, and the 10q26 ARMS2/HTRA1.^10^^,^^12^, ^13^, ^14^, ^15^, ^16^, ^17^ Additionally, large genome-wide association studies by an international consortium have identified 34 loci associated with AMD at a genome-wide significance level.^10^ Although certain risk factors for AMD such as cigarette smoking and dietary intake of antioxidants and fatty acids are widely accepted, others are more controversial, such as sunlight exposure, cardiovascular risk factors, alcohol consumption, etc.

In recent years, an infectious hypothesis in the pathogenesis of AMD has emerged, suggesting a link between various infectious agents and AMD. This hypothesis stems mainly (1) from the implication of low-grade inflammation in AMD and (2) from the knowledge that infection can exacerbate the inflammatory response. In the fields of infectious diseases and immunology, there is a growing body of research linking chronic low-level infections to diseases that are not typically thought of as infectious. In particular, chronic bacterial infections (e.g., *Chlamydia pneumoniae* [*C.* *pneumoniae*], *Helicobacter pylori* [*H. pylori*]) have been discussed to be linked to conditions like atherosclerosis and cancer.^18^, ^19^, ^20^ This has led to a greater awareness that chronic or latent infections could still have long-term consequences for tissue health, including those of the eye. In neurodegenerative diseases like Alzheimer’s disease and Parkinson’s disease, several infections have also been suggested to play a role in triggering or accelerating neurodegeneration.^21^^,^^22^ This idea has been extended to the retina, which is considered part of the central nervous system. In order to assess the current state of knowledge on the diverse pathogens studied (including bacteria, yeasts, and viruses), we decided to conduct this scoping review. Our objectives were to investigate which pathogens had been studied, using which methodology, and which underlying pathophysiological mechanisms were suspected. Ultimately, we wanted to assess the current level of evidence for the infectious hypothesis in AMD and identify potential avenues for further research.

## Methods

### Eligibility Criteria for Considering Studies for This Review

Following the PRISMA scoping review guidelines,^23^ we conducted a scoping review aiming to identify articles in English assessing the potential involvement of infectious agents in AMD.

- In vitro, animal, and human studies (including postmortem studies) as well as hypothesis papers were considered. Literature reviews and editorials were excluded.
- All types of conventional infectious agents were considered including bacteria, viruses, fungi (whatever the diagnostic method) as well as various proxies (e.g., pathogen free environment versus conventional open environment). Nevertheless, given that recent high-quality literature reviews already existed on oral and gut microbiota,^24^, ^25^, ^26^, ^27^ we decided not to include the related articles in this review. In addition, an article dealing with the impact of the recent severe acute respiratory syndrome coronavirus type 2 (SARS-COV2) pandemic on AMD was also excluded because it appeared that our algorithm did not allow for the proper identification of studies involving SARS-COV2 in AMD. Indeed, existing studies investigate the consequences of SARS-COV2 infection on all ocular structures (not specifically AMD) and, consequently, AMD-related terms were not systematically present in their title and/or abstract. Finally, our algorithm was not designed to identify studies investigating only certain bacterial components (e.g., lipopolysaccharide [LPS]), frequently used to study the impact of inflammation; we therefore excluded these studies as well.
- In human studies, all AMD stages were considered and AMD diagnoses could be based on clinical or medico-administrative data. In in vitro and animal studies, all types of AMD-related lesions (e.g., drusen, CNV, etc) were considered.

### Search Methods for Identifying Studies

We searched the PubMed database for articles in English published up to June 1, 2023, without any temporal or geographical limitations. Using a search algorithm to find articles whose title and/or abstract contain a combination of terms related to (1) AMD and (2) infectious agents (details in Appendix 1 available at https://www.ophthalmologyscience.org), we identified 868 articles related to our topic of interest. The list of records identified was exported into the reference management software Zotero 7 (Corporation for Digital Scholarship) and into an Excel table (Microsoft Corporation) to assess eligibility criteria. Subsequently, we also screened the reference lists of selected studies.

No institutional board review/ethics committee approval was required for this scoping review.

### Study Selection

**Figure 1:**
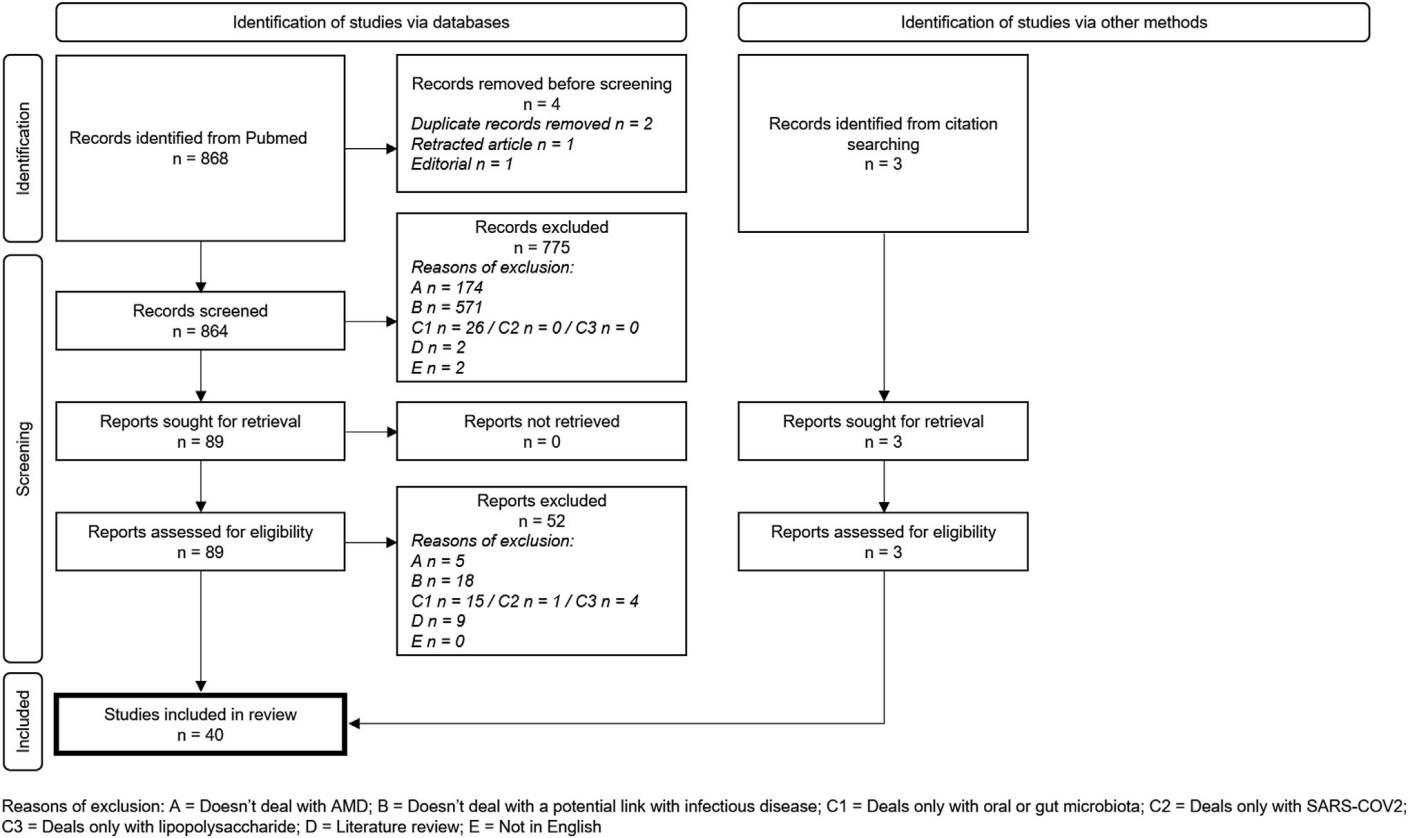
Flow chart illustrating the article identification and screening process. AMD = age-related macular degeneration; SARS-COV2 = severe acute respiratory syndrome coronavirus type 2.

After removing duplicates (n = 2), a retracted article (n = 1), and an editorial (n = 1), 2 investigators (L.P. and L.M.) independently screened the 864 remaining references and then assessed 89 reports for eligibility (flow chart in Fig 1). To note, 2 articles in Russian^28^^,^^29^ could not be included, although they dealt with our subject of interest. Three additional records were identified from citation searching using the reference lists of selected studies and assessed for eligibility. Discrepancies were resolved through discussion between the investigators. In total, 40 articles were included in our review (see the list of included articles in Table S1 available at https://www.ophthalmologyscience.org).

### Data Collection

From the selected articles, we systematically extracted the following items in calibrated Excel tables.

- For human studies, we extracted the following: first author, title, journal, year of publication, infectious agent(s) investigated, data source (including location, type of study, baseline, or study period if available), study design, participants (including number and main inclusion/exclusion criteria), diagnostic methods for both infection and AMD, statistical methods, and main results.

- For experimental studies, we extracted the following: first author, title, journal, year of publication, infectious agent(s) investigated, type of study, cell line studied or used animal models, exposure protocol for the infectious agent in question, main outcomes, and results.

L.P. extracted data for studies involving bacteria or fungi and L.M. for viruses or infectious proxies. The extraction was then checked by the second investigator.

To note, for the classification of AMD, several internationally accepted classifications exist.^8^, ^9^, ^10^ To ensure comparability and consistency within this article, we used the widely accepted Beckman classification and harmonized the terminology of included studies. Of note, according to the Beckman classification, the term dry AMD is only used for GA. Therefore, the term nonexudative AMD was used to summarize early AMD, intermediate AMD, and GA. We are aware that in light of new insights learned from OCT angiography (OCTA), also the term nonexudative may be not entirely unambiguous due to the existence of nonexudative CNV.^11^ However, most of the included studies were performed in the pre-OCTA era, and none of the included studies applied OCTA, indicating that all studies included in this scoping review refer to exudative CNV when using the term CNV.

### Data Synthesis

The results of each of the 40 studies are detailed in Tables S2 and S3 (available at https://www.ophthalmologyscience.org) and summarized by pathogen type in the Results section below. An overview of the studies is also presented in Table 4. Moreover, in order to present a relatively complete overview of the topic, a brief summary of previously published literature reviews on the oral and gut microbiota is also presented at the end of the Results section.

**Table 4:** Brief Overview of the Main Results of the 40 Studies Identified

	Human Studies	Studies on Animal, In Vitro or Other	Total∗
Chlamydia pneumoniae (Cp)	**Association between *Cp* serostatus and AMD (n = 11):** Seropositivity to *Cp*: []^31^ □^41^ []^42^ □^46^^43^ Levels of anti-*Cp* antibodies: ▪^33^^35^ []^36^^37^ []^38^^40^ []^42^^43^ **Search for *Cp* in human tissues (n = 4):** PCR study using peripheral blood cells: ^47^ PCR/IHC studies using ocular tissues: ^47^^34^^39^^32^	**In vitro or animal studies (n = 3):** Human *Cp*-infected macrophages and RPE cells had an increased production of VEGF and inflammatory cytokines, respectively^34^ Murine RPE cells in contact with *Cp* antigen or with LPS secreted IL-6 and VEGF. Intravitreous injection of the *Cp* antigen led to increased sizes of laser-induced CNV compared to controls and to increased secretion of IL-6 and VEGF in the intraocular fluid^44^ Mice intraperitoneally infected with *Cp* or exposed to low doses of LPS had increased size of laser-induced CNV compared to controls. Prior exposure to low doses of LPS induced a long-term epigenetic reprogramming of retina-resident microglia and subsequent reduced inflammatory but enhanced angiogenic phenotypes.^45^	17

Nasal or pharyngeal microbiota	**Investigation of the microbiota using 16s rRNA gene sequencing (n = 2):** Nasopharynx microbiota: []^51^ Nasal microbiota: ^52^		2

Other bacteria and yeasts	**Association between certain bacteria and AMD (n = 4):** *Chlamydia trachomatis:* □^46^ □^33^ *Chlamydia psittaci:* □^46^ *Escherichia coli:* □^33^ *Mycoplasma pneumoniae:* []^42^ *Helicobacter pylori:* []^36^ **Case report (n = 1)** suggesting an impact of *M. chelonae* on progression to neovascular AMD*:*^53^ **Search for *B. megaterium* in soft drusen (n = 1)**: ^54^	**Animal studies (n = 2):** Drusen-like pathology after injection of *B. megaterium* in a nonhuman primate model ^54^ Systemic exposure to *Candida albicans* led to retinal microglial activation in mice ^55^	6

HIV	**Association between HIV and AMD (n = 2) using**: Clinical diagnosis: ^56^^57^		2

Hepatitis B virus (HBV)	**Association between HBV and AMD (n = 4) using:** Serostatus: •^60^^61^ Medicoadministrative data: ♦^62^ []^63^	**In vitro study (n = 1):** HBV could sensitize RPE cells to UV or blue light-induced cell death through downregulation of DNA repair pathways^62^	4

Hepatitis C virus (HCV)	**Association between HCV and AMD (n = 4) using:** Serostatus: ○^60^ Serostatus and coinfection in patients with HIV: ○^56^^57^ Medico-administrative data: []^76^		4

Varicella-zoster virus (VZV)	**Association between VZV and AMD (n = 1) using:** Diagnosis of herpes zoster ophthalmicus in medico-administrative data: ^30^		1
Cytomegalovirus (CMV)	**Association between CMV and AMD (n = 1) using:** Levels of anti-CMV antibodies: []^36^ **Anatomopathological study (n = 1)** searching for CMV DNA in posterior eye cups of human cadavers without AMD: ▴^90^	**In vitro or animal studies (n = 5):** Macrophages infected with murine CMV were associated with increased levels of proangiogenic factors, consistent with an M2 phenotype. Systemic murine CMV infection led to larger laser-induced CNV lesions related to the activation of CMV-infected macrophages toward a proangiogenic M2 phenotype.^89^ Following systemic infection, murine CMV can establish a latent infection within the choroid and in few RPE cells. Latent murine CMV infection was associated with increased levels of inflammatory and proangiogenic factors as well as with retinal and choroidal pathologies. 90, 91, 92 Systemic murine CMV infection led to an increased number of activated microglial cells within the subretinal space.^93^	6

Human herpes virus 6A (HHV-6A)	**Hypothesis paper (n = 1) on** HHV-6A and AMD:^94^		1

Other markers of infection	History of lung infection ♦^97^ Specific microbial signature in the aqueous humor of patients with AMD ▪^100^	**In vitro or animal studies (n = 2):** Complement factor H (CFH) knock out mice exposed to a conventional open environment had higher number of subretinal macrophages, higher levels of proinflammatory cytokines and stress markers and a reduced number of photoreceptors, compared to CFH knock out mice exposed to a pathogen free environment.^98^ Stimulation of human RPE cells with viral double-stranded RNA analog induced the expression of the angiogenic factors, inflammatory cytokines, and complement proteins. Conversely, exposition to viral/bacterial DNA analog had a lesser impact on gene expression.^99^	4

Total∗	30	13	40


Study design and conclusions: □■ Case-control ○● Cross-sectional ◊♦ Cohort or nested case-control Δ▲ Other study design. Filled or empty shapes for results against and in favor of a link between infections and AMD, respectively.

AMD stages investigated: Black: all stages of AMD; Light blue: early AMD; Dark blue: progression from early AMD; Purple: intermediate AMD; Green: nonexudative AMD; Pink: advanced AMD; Orange: geographic atrophy; Red: neovascular AMD.

When, in the same study, results are available for different stages of AMD or for different designs, they are presented in square brackets.

AMD = age-related macular degeneration; *B. megaterium* = Bacillus megaterium; CMV = *Cytomegalovirus*; CNV = choroidal neovascularization; DNA = deoxyribonucleic acid; HIV = human immunodeficiency virus; IHC = immunohistochemistry; IL = interleukin; LPS = lipopolysaccharide; *M. chelonae* = *Mycobacterium chelonae*; PCR = polymerase chain reaction; RPE = retinal pigment epithelium; rRNA = ribosomal ribonucleic acid; VZV = varicella-zoster virus.

∗ Some studies concern several pathogens and use several study methods.

Potential pathophysiological mechanisms are discussed (1) pathogen by pathogen, according to the elements evoked in each of the articles in the Results section and (2) more globally in the Discussion section.

## Results

After screening 868 articles, 89 were assessed in full for eligibility (detailed flow chart in Fig 1). In total, we included 37 original articles or letters in English dealing with the potential impact of infectious agents in AMD. Reviewing the reference lists of all selected articles, we identified 3 additional articles that were not captured by our research algorithm (one letter to the editor on varicella-zoster virus [VZV]^30^ and 2 studies on *C. pneumoniae*^31^^,^^32^). Finally, 40 articles were included in our review. The results of these studies are detailed in Tables S2 and S3 (available at https://www.ophthalmologyscience.org) and briefly presented below by pathogen type (see also Table 4 for an overview).

Briefly, the results described in these 40 studies were obtained from human data (n = 30), animal (n = 9) or in vitro (n = 6) experiments (sometimes with several data types in the same article), and one hypothesis paper. The studies focused on either bacteria (n = 21), fungi (n = 1), viruses (n = 15), or other biomarkers of infections (n = 4).

### Studies Included in Our Review

#### Chlamydia Pneumoniae

We identified a total of 17 original studies on *C. pneumoniae* and AMD.^31^, ^32^, ^33^, ^34^, ^35^, ^36^, ^37^, ^38^, ^39^, ^40^, ^41^, ^42^, ^43^, ^44^, ^45^, ^46^, ^47^ Among them, 12 studies investigated the potential associations between *C. pneumoniae* serological status and either AMD (n = 10) or its progression (n = 2). Four studies searched for the presence of *C. pneumoniae* within human eye tissues and 3 were experimental studies (in vitro and/or on animal models). Of note, one study included both serological and histopathological data, and another study included both histopathological and experimental data.

Five studies explored the potential association between seropositivity to *C. pneumoniae* and the prevalence of AMD.^31^^,^^41^, ^42^, ^43^^,^^46^ Among these studies, 3 were case-control studies,^41^^,^^42^^,^^46^ one a nested case-control study,^31^ and one was a cross-sectional study.^43^ Of note, in the nested case-control study, the authors also investigated the impact of *C. pneumoniae* infection on the incidence or progression of AMD after 10 years. None of the case-control studies found a significant association between *C. pneumoniae* seropositivity and AMD. Associations were also nonsignificant in studies analyzing early AMD, GA, or neovascular AMD separately.^31^^,^^42^^,^^46^ Conversely, the cross-sectional study by Nakata et al (which benefited from a larger sample size of 971 subjects with large drusen and 3209 without) reported a significant association between *C. pneumoniae* seropositivity and intermediate AMD despite relatively small differences in seroprevalence (56.4% in subjects with intermediate AMD vs. 51.7% in those without; *P* = 0.01). One study^46^ also investigated potential interactions with genetic risk factors: it highlighted that, among carriers of CFH Y402 risk *C.* *allele*, seropositivity for *C. pneumoniae* differed slightly between early and advanced AMD (*P* = 0.09, 60.0% of infected subjects in early AMD vs. 75.7% in advanced AMD) whereas there was no difference between controls and pooled AMD cases (70.1% and 72.2%, respectively).

Eight studies explored the potential association between the levels of anti *C. pneumoniae* immunoglobulin G (IgG) (which may reflect more intense exposure to *C. pneumoniae* [i.e. higher concentrations of bacteria as well as chronic or recurrent infections]) and the prevalence of AMD (n = 6) or its progression (n = 2). Among these, 4 case-control studies^33^^,^^35^^,^^36^^,^^42^ found mixed results. The first 2 studies highlighted significantly increased levels of anti *C. pneumoniae* IgG in subjects with any AMD^33^ or neovascular AMD^35^ compared with controls. The next 2 studies^36^^,^^42^ found no statistically significant association between these levels of anti *C. pneumoniae* IgG and AMD, studying nonexudative AMD (i.e., early AMD, intermediate AMD, or GA), neovascular AMD, and controls separately. All these 4 studies suffered from small study samples of less than 100 cases and limited adjustment. The 2 remaining studies on anti *C. pneumoniae* IgG and the prevalence of AMD had larger study samples with wide adjustment.^38^^,^^43^ The cross-sectional study by Nakata et al revealed a significant association between anti *C. pneumoniae* IgG levels and the prevalence of intermediate AMD, indicated by the presence of drusen ≥ 125 μm (adjusted odds ratio [aOR] = 1.020 95% confidence interval [95%CI] [1.001–1.040]; *P* < 0.05). The nested case-control study by Robman et al found no significant association between levels of anti *C. pneumoniae* IgG and any prevalent AMD (n = 197 cases of AMD, aOR = 1.02 95%CI [0.66–1.56] comparing the third tertile to the first tertile of IgG), early AMD (n = 159 cases, aOR = 0.97 95%CI [0.61–1.53]), or advanced AMD (n = 38 cases, aOR = 1.23 95%CI [0.52–2.92]). Nevertheless, given the limited number of advanced AMD cases, a differential impact of the infection depending on the AMD stage remains to be investigated. Finally, 2 studies performed within the prospective “Cardiovascular Health and Age-related Maculopathy Study” explored the association between levels of IgG and the progression of AMD (using a 6-level severity scale and 3 different definitions of grading) in a cohort of participants with early AMD.^37^^,^^40^ They highlighted that subjects in the second and the third tertile of anti *C. pneumoniae* IgG titers had a significantly increased risk of 7-year AMD progression compared with those in the first tertile (aOR = 2.10 95%CI [1.00–4.50] and aOR = 2.58 95%CI [1.24–5.41], respectively). Moreover, they hypothesized a potential interaction between infection by *C.* *pneumoniae* and the CC risk genotype of the CFH gene. In multivariate models, although the CC genotype was associated with an increased risk of AMD progression (aOR = 2.43 95%CI [1.07–5.49], *P* = 0.03, compared to the TT genotype), having both the CC genotype and being within the upper tertile of antibodies to *C. pneumoniae* was associated with a greater risk of AMD progression (aOR = 11.8 95%CI [2.1–65.8], *P* = 0.005, compared to TT genotype within the lowest tertile).

Regarding the underlying mechanisms, several hypotheses have been discussed. Firstly, *C. pneumoniae* is an obligate intracellular bacterium which can infect the respiratory tract and establish persistent infection in humans and subsequent chronic inflammation. Such chronic systemic inflammation has been proposed to have an impact at sites distant from the infection and could therefore contribute to diseases with an inflammatory component such as AMD. To note, a role of *C. pneumoniae* has been suggested in several other inflammatory diseases (including atherosclerosis) but remains controversial.^18^^,^^48^ Exposure to peripheral immune stimuli (such as *C. pneumoniae* or the endotoxin LPS, which is part of its outer membrane) has also been suggested to provoke a long-term reprogramming of immune cells at distant sites (including retina-resident microglia), which could have detrimental effects upon subsequent immune stimuli, potentially contributing to the onset of AMD.^45^ Secondly, infection of macrophages by *C. pneumoniae* allows dissemination of *C. pneumoniae* to various organs including ocular tissues and especially the choroid (given the particular attraction of *C. pneumoniae* for vascular tissue). Subsequently, *C. pneumoniae* may infect various cells types, including RPE cells (which are the first line of defense against external pathogens by maintaining of the blood–retina barrier) or retinal microglial cells. Once in the eye, *C. pneumoniae* could promote AMD through various mechanisms including local chronic secretion of inflammatory cytokines and chemokines (and subsequent recruitment of immune cells^49^), the secretion of proangiogenic factors or an induction of lipoprotein oxidation, dysregulation of cellular lipid metabolism, and dysregulation of apoptotic pathways, all mechanisms which have been implicated in AMD pathogenesis.^33^^,^^34^ Because the most common trigger of the alternative pathway are microbial in nature, *C. pneumoniae* could also lead to uncontrolled complement activation, in particular in subjects carrying a risk variant of the CFH gene.^38^^,^^50^

Several studies have provided clues as to the mechanisms potentially involved, analyzing the presence of *C. pneumoniae* in human tissues and/or blood. In 2009, one study^47^ analyzed the presence of *C. pneumoniae* DNA in peripheral blood cells by polymerase chain reaction (PCR), highlighting a higher prevalence of infection in subjects with advanced AMD (n = 148; 20.3%) than in controls (n = 162; 10.5%; *P* = 0.02). Conversely, PCR analyses of microdissected macular cells of paraffin-embedded ocular slides showed the presence of *C. pneumoniae* in only a small number of samples: 2 out of 59 advanced AMD samples (GA or neovascular) (3.4%) and one out of 16 (6.3%) age-matched control samples (the difference was not statistically significant). Three studies analyzed the presence of *C. pneumoniae* in CNV, using surgically excised CNV specimens of patients with AMD. These CNV samples were excised in the pre-anti-VEGF era, when surgical removal of CNV used to be a valid therapy option. In 2005,^34^ one study reported *C. pneumoniae* in 4 out of 9 (44.4%) and 2 out of 9 (22.2%) AMD CNV paraffin-embedded specimens, using immunohistochemistry (IHC) and PCR, respectively. Conversely, *C. pneumoniae* was not found in any of the 21 control samples (including in particular 5 non-AMD CNV specimens and 16 non-AMD eyes). In 2006, however, one study^39^ using 13 AMD CNV frozen specimens did neither detect *C. pneumoniae* DNA by PCR, nor any other bacterial DNA using a broad range bacterial 16S ribosomal DNA PCR. In 2013, one study using IHC^32^ detected *C. pneumoniae* in 17 out of 25 (68.0%) AMD CNV paraffin-embedded specimens. Notably, macrophages were also visualized in 19 specimens including 16 positive to *C. pneumoniae* using IHC. These discrepancies between studies may be explained by the use of different methodologies (PCR vs. IHC) and pretreatments of analyzed tissues. To note, the detection of *C. pneumoniae* might have been underestimated (1) in PCR studies using formalin-fixed tissues (as formalin degrades DNA)^34^ and (2) due to a patchy distribution of the infection.^32^^,^^34^ One of the studies using IHC showed that on average only 6% (varying from 1% to 18%) of the cells were infected by *C. pneumoniae,* indicating a strong influence of sampling on the results.^32^ However, neither the absence of *C. pneumoniae* excludes its pathological role (i.e., the "hit and run hypothesis" suggests a secondary elimination of *C. pneumoniae* after damage has occurred), nor does its presence confirm its causal role (i.e., the "innocent bystander hypothesis" suggests that pre-existing inflammation could promote the recruitment of *C. pneumonia-*infected monocytes to already damaged ocular tissues).

Several in vitro and animal studies have also investigated some of the potential underlying mechanisms. In 2005, *C. pneumoniae* was shown to infect both human macrophages and RPE cells in vitro. Moreover, it was reported that the infection increased the production of VEGF by human macrophages and that of interleukin (IL)-8 and monocyte chemoattractant protein-1, both inflammatory chemokines implicated in the pathogenesis of AMD, by RPE cells.^34^ In 2010,^44^ a study showed that the inoculation of *C. pneumoniae* antigen to primary-cultured murine RPE cells led to a toll-like receptor 2 dependent secretion of IL-6 (multifunctional inflammatory cytokine promoting both angiogenesis and host defense against pathogens) and VEGF. Notably, stimulation with LPSs also led to the secretion of IL-6, VEGF, and tumor necrosis factor (TNF)α by RPE cells. Consistently, using C57BL/6 mice, intravitreous injection of the *C. pneumoniae* antigen was shown to increase the size of laser-induced CNV and to increase secretion of IL-6 and VEGF in the intraocular fluid compared to controls. The use of different knockout mouse models also confirmed the toll-like receptor 2-dependent mechanism. In 2023^45^, using intraperitoneally infected C57BL/6J mice, Hata et al confirmed the larger size of laser-induced CNV in infected mice compared to controls. Subsequently, they investigated the impact of *C. pneumoniae* infection through exposure to LPS showing that laser treatment performed 4 weeks after exposure to low doses of LPS led to more pronounced CNV in LPS-exposed mice than in controls. Further experiments led them to conclude that prior peripheral exposure to low doses of LPS induced a long-term epigenetic reprogramming of retina-resident microglia and subsequent reduced inflammatory but enhanced angiogenic phenotype, which may contribute to the development of CNV lesions. The apparent discrepancy between proinflammatory and anti-inflammatory properties of *C. pneumoniae* may be explained by differences in experimental conditions (in vitro, in vivo, the timing, the intensity, and the location of exposure).

#### Nasal and Pharyngeal Microbiota

In 2018, a case-control study from Singapore investigated the differences of microbial colonization of the human nasopharynx between AMD patients (both early and advanced AMD) and controls, using 16s ribosomal RNA gene sequencing.^51^ Although the alpha-diversity (bacterial variation within a single sample) indices and the global composition of the microbiome were similar between AMD patients (n = 245) and controls (n = 386), subtle differences in relative abundance for some bacterial genera were highlighted. Generalized Linear Mixed Model analysis revealed that AMD cases had increased relative abundance of *Gemella* (6.0% vs. 4.0%, adjusted *P* = 0.007) and *Streptococcus* (23.4% vs. 18.6%, adjusted *P* = 0.002) and decreased relative abundance of *Prevotella* (12.7% vs. 19.3%, adjusted *P* = 6.95E−5) and *Leptotrichia* (0.8% vs. 1.4%, adjusted *P* = 0.007) compared with controls. Comparing advanced AMD to controls produced similar results while early AMD did not differ from controls, suggesting potential dysbiosis in advanced stages of the disease. Analyzing the subset of patients >60 years old revealed significantly different relative abundances for the *Prevotella*, *Leptotrichia*, and *Streptococcus* genera in AMD cases compared to controls.

In 2020, a small study from Canada^52^ published data on nasal microbiota and its association with neovascular AMD, also using 16s ribosomal RNA gene sequencing. Some bacterial communities had an increased relative abundance in AMD cases (n = 13) compared to controls (n = 5) including the *Burkholderiales* order (sevenfold higher; *P* = 3.29E-05), the *Actinomycetaceae* family (sixfold higher; *P* = 3.73E-06), the *Gemella* genus (fivefold higher; *P* = 0.0002), the *Proteobacteria* family (fourfold higher; *P* = 0.004), the *Actinomyces* species (threefold higher; *P* = 0.002), the *Streptococcus* species (threefold higher; *P* = 0.01), and the *Veillonella* species (twofold higher; *P* = 0.005). Conversely, members of the *Clostridia* class were fourfold higher in controls (*P* = 0.007).

Regarding potential underlying mechanisms, the authors hypothesize that changes in the composition of nasal and pharyngeal microbiota could lead to local inflammation and increased mucosal permeability. Subsequently, this could facilitate transmission of bacteria or the dissemination of proinflammatory products to distant sites (such as the eye) inducing inflammation.

#### Other Bacteria and Fungi

##### Mycobacterium Chelonae

In 2016, a rare case of subacute bilateral transformation from nonexudative to neovascular AMD associated with bilateral choroiditis was described in a patient of about 70 years of age.^53^ The authors hypothesized a possible involvement of *Mycobacterium chelonae* (*M. chelonae*), given (1) a recent episode of lung infection due to *M. chelonae* and (2) a history of autoimmune disease (which can favor the hematogenous dissemination of the bacteria). They suggested that mycobacterial dissemination may have induced bilateral choroiditis, triggering a local proangiogenic response, which may have resulted in progression to neovascular AMD.

##### Bacillus Megaterium

In a review from 2018^54^, the authors mentioned unpublished data highlighting (1) an enrichment with *Bacillus megaterium* (*B. megaterium*) in soft drusen in patients with AMD and (2) an induction of a drusen-like pathology after subretinal injection of *B. megaterium* in a nonhuman primate model. No mechanistic details were reported, and, to our knowledge, the complete data are still unpublished.

##### Other Bacteria

Several studies mainly focusing on *C. pneumoniae* also reported results on other types of bacteria. A case-control study^33^ found similar levels of IgG directed against antigens of *Chlamydia trachomatis* (*C. trachomatis*) and *Escherichia coli (E. coli)* comparing 25 AMD cases and 18 controls. Another case-control study^42^ found no significant differences between the levels of IgG directed against *Mycoplasma pneumoniae (M. pneumoniae)* in subjects with neovascular AMD (n = 20), nonexudative AMD (n = 20), and age- and sex-matched controls (n = 20). In a third case-control study,^46^ including 199 patients with AMD and 100 controls, seropositivity to *C. trachomatis* or *Chlamydia psittaci* (*C. psittaci)* was not significantly associated with AMD. Finally, Miller et al^36^ found similar levels of IgG directed against *H. pylori* comparing neovascular AMD (n = 47), nonexudative AMD (n = 36), and controls (n = 67).

##### Candida Albicans

In 2014, using C57BL/6J mice injected intravenously with a sublethal dose of Candida albicans (*C. albicans*), the authors highlighted that a systemic infection with *C. albicans* can cause (1) an increase in the number of microglial cells in the retina, (2) microglia activation, and (3) microglial relocation within the retinal layers.^55^ Regarding the underlying mechanisms, *C. albicans* was not detected in retinal tissue. Thus, the authors suggested that microglia activation was because of either a high cytokine production secondary to systemic infection or to a direct interaction between microglia and soluble fungal ligands. The authors hypothesized that excessive or prolonged activation of microglia may subsequently lead to chronic retinal inflammation and subsequent retinal damage.

#### Human Immunodeficiency Virus

Using data from the American “Longitudinal Study of the Ocular Complications of AIDS” (LSOCA), Jabs et al^56^ found a prevalence of 9.9% of intermediate-stage AMD (graded by retinal photographs at inclusion) among 1825 patients with AIDS and without ocular opportunistic infections. The comparison to the previously published cohort of 2810 human immunodeficiency virus (HIV)-uninfected subjects of the Beaver Dam Offspring Study highlighted a threefold increase in crude prevalence of AMD for AIDS patients (9.9% vs. 3.3%) and a fourfold increase after adjustment for age and sex. Subsequently, the authors also compared the incidence of intermediate-stage AMD at the 5- and 10-year follow-ups in the LSOCA study (730 and 379 participants, respectively) to that estimated among approximately 3685 HIV-uninfected participants of the Multi-Ethnic Study of Atherosclerosis (MESA).^57^ Patients with AIDS had an increased risk of intermediate-stage AMD (relative risk = 1.75 95%CI [1.16–2.64]; *P* = 0.008 after adjustment for ethnicity and sex) although HIV-uninfected subjects of the MESA cohort were older than those included in the LSOCA cohort (61 ± 9 vs. 44 ± 8 years). Thus, the authors hypothesized that the increased risk of AMD seen in patients with AIDS was linked to the impact of HIV on the immune system. Indeed, HIV infection has been associated to chronic systemic inflammation, chronic immune activation, and a certain degree of immunosenescence,^58^^,^^59^ components that could influence the risk of AMD. Moreover, as mentioned by the authors, their results could also be explained by selection bias. Because all participants in the LSOCA study were enrolled at AIDS ophthalmology clinics, they were more likely to have ocular diseases than participants in the Beaver Dam Offspring Study and the MESA study, which were population-based studies.

#### Hepatitis B Virus

In 2008, using data from a South Korean cross-sectional study^60^ including 9530 participants >40 years of age, Roh et al evaluated potential risk factors for AMD. Age-related macular degeneration was diagnosed by fundus photography in 235 (2.5%) subjects, of which 215 had early AMD. Carriers of hepatitis B surface antigen (measured in the serum of patients and reflecting present hepatitis B virus [HBV] infection) had a twofold increased risk of AMD compared with uninfected subjects (aOR = 2.56 95%CI [1.48–4.42] after adjusting for known risk factors of AMD including sociodemographic status). Subsequently, another South Korean study^61^ including 14 352 participants above the age of 40 years found similar results in 2014: hepatitis B surface antigen carriers had a twofold increased risk of early AMD compared with uninfected subjects (aOR = 1.98 95%CI [1.38–2.85], *P* < 0.001). Although no significant association was found between HBV infection and advanced AMD (aOR = 1.49 95%CI [0.51–4.32], *P* = 0.47, after adjustment for age, sex, and smoking status), this analysis appears to suffer from low statistical power (only 5 infected subjects with advanced AMD).

More recently, 2 prospective studies^62^^,^^63^ based on the Taiwanese Longitudinal Health Insurance Database 2000 have also investigated associations between HBV and either macular degeneration or AMD. Unlike the 2 previous studies, which benefited from systematic screening, diagnoses of HBV and macular degeneration were based on information available in the health insurance database leading to a risk of underdiagnosis for both pathologies. Nevertheless, this limitation is partly counterbalanced by the very large size of the samples. The first study included 39 796 subjects with HBV (whether acute or chronic) over the age of 20 and 159 184 age-matched controls and highlighted that infected subjects had an increased risk of macular degeneration (adjusted hazard ratio [aHR] = 1.31 95%CI [1.17–1.46] after adjustment for age, sex, and some comorbidities but not tobacco consumption). After dividing the study population into 3 age groups (≤34, 35–49, and ≥50 years), this association remained statistically significant only in subjects >50 years of age (aHR = 1.24 95%CI [1.10–1.40]). Subsequently, a second analysis of these data was carried out focusing on subjects with chronic HBV infection. Using data of 17 796 subjects over the age of 40 with chronic HBV infection and 71 184 age and sex-matched controls, an increased risk of AMD (aHR = 1.41 95%CI [1.23–1.63], *P* < 0.001, after adjustment for age, sex, socioeconomic factors, several comorbidities but not tobacco consumption) was observed. Associations remained statistically significant when focusing on nonexudative AMD (aHR = 1.41 95%CI [1.19–1.65], *P* < 0.001), neovascular AMD (aHR = 1.43 95%CI [1.08–1.89], *P* = 0.01), and the progression from nonexudative to neovascular AMD (aHR = 1.74 95%CI [1.01–2.99], *P* = 0.01). Although these analyses appear to confirm the results obtained in the South Korean studies, it should be noted that a follow-up bias cannot be excluded (i.e. subjects with HBV infection are probably more frequently followed ophthalmologically because of HBV, its treatment or to a higher frequency of related comorbidities), and therefore have a greater likelihood of being diagnosed with AMD.

Regarding the underlying pathophysiological mechanism, several hypotheses have been discussed by the authors

- Firstly, Chou et al^62^ investigated a potential direct impact of HBV on RPE cells. In vitro, they induced an overexpression of HBx protein, the pathogenic X protein of HBV using ARPE-19 cells (a mainly immortalized human RPE cell line). Using ultraviolet (UV) and blue light exposures to induce oxidative stress and subsequent cellular damage, they showed that HBx-transfected cells had reduced cell viability and reduced clonogenic survival compared to mock-transfected cells. This suggests that HBV infection could sensitize RPE cells to UV or blue light-induced cell death. Furthermore, differentially expressed genes between HBx-transfected and mock-transfected cells are described both before and after UV exposure, indicating a downregulation of various DNA repair pathways by HBx. Nevertheless, although HBV biomarkers are frequently reported in tears, aqueous humor,^64^, ^65^, ^66^, ^67^ cornea, or lenses,^68^^,^^69^ the presence of HBV within AMD-affected tissues remains unclear. If hepatitis B surface antigen was detected in the subretinal fluid of an HBV-positive patient,^70^ to our knowledge, no study has investigated the presence of HBV in RPE cells or in retinal samples.

- Secondly, a more indirect mechanism has also been suggested: HBV-induced antibodies or effector lymphocytes could cross-react with the retinal S-Ag, a major component of photoreceptor cells, which has sequence homology with the HBV DNA polymerase,^71^ and thus lead to subsequent inflammation and/or tissue damage (even in the absence of the virus within the eye). Although the existence of an autoimmune component in the development of AMD remains debated, elevated levels of retinal autoantibodies were described in patients with AMD compared to controls^72^^,^^73^ and, interestingly, higher rates and titers of autoantibodies against retinal S-Ag were also described among HBV healthy carriers compared to controls.^74^

- Finally, HBV infection may also indirectly activate the alternative complement pathway, which is an important player in the development of AMD. Indeed, in vitro, HBx protein has been shown to downregulate the CFH (an inhibitor of the alternative complement pathway).^75^

#### Hepatitis C Virus

In 2021, another study^76^ based on the Taiwanese Longitudinal Health Insurance Database 2000 investigated the risk of incident AMD in 13 300 subjects over 18 years old with a diagnosis of chronic hepatitis C virus [HCV] infection and 26 600 propensity score-matched subjects without HCV. Subjects coinfected with HBV or HIV were excluded. The propensity score included age, sex, socioeconomic characteristics, and some comorbidities. An increased risk of AMD was observed among HCV-infected subjects (aHR = 1.22 95%CI [1.09–1.35], *P* < 0.001) compared to controls. The association remained significant for nonexudative AMD (aHR = 1.22 95%CI [1.09–1.37], *P* < 0.001) but not for neovascular AMD (aHR = 1.17 95%CI [0.84–1.61], *P* = 0.35). Because interferon therapy is known to induce retinopathy,^77^^,^^78^ the incidence of AMD was compared between 1973 HCV-infected subjects treated by pegylated interferon and ribavirin and 3946 HCV-infected untreated subjects matched by a propensity score (including age, sex, socioeconomic factors, and some comorbidities). No statistically significant difference was found between being treated and AMD (aHR = 1.07 95%CI [0.81–1.43], *P* = 0.24) or its subtypes (neovascular AMD: aHR = 1.36 95%CI [0.58–3.22], *P* = 0.497; nonexudative AMD: aHR = 1.04 95%CI [0.77–1.41], *P* = 0.78). Based on these results, the authors conclude that the increased risk of AMD is probably linked to HCV infection itself and not to treatment by pegylated interferon and ribavirin. Certain limitations (previously mentioned for other studies carried out on this database) should be considered: a risk of underdiagnosis because of the absence of active screening for both pathologies, the lack of information on smoking or genetics, and a risk of follow-up bias increasing the probability of a diagnosis of AMD in subjects followed for HCV infection.

Three other studies investigating the role of HCV infection reported negative results.^56^^,^^57^^,^^60^ In a population of patients with AIDS included in the LSOCA cohort (2 studies by Jabs et al previously described in the HIV section), being coinfected with HCV was not associated with an increased prevalence^56^ or incidence^57^ of intermediate-stage AMD. Using data from a South Korean cross-sectional study (previously described in the HBV section), Roh et al^60^ also reported a lack of association between HCV infection and AMD. However, this result suffers from low statistical power (only 1 HCV-infected subject with AMD).

Regarding the underlying pathophysiological mechanism, few elements have been discussed. It has been suggested that HCV could act directly within the eye. Indeed, HCV is known to infect certain compartments of the eye including the tears, the cornea, and the aqueous humor^79^ and has also been associated with various ophthalmological disorders,^79^ including retinal damage (retinal pigment epithelitis,^80^ ischemic retinopathy^77^). However, no information is available on the precise mechanism of action, which may involve the virus itself or the immune response to it.

#### Varicella-Zoster Virus

In 2019, using data from the Taiwanese Longitudinal Health Insurance Database 2005, Ho et al^30^ analyzed the associations between the onset of herpes zoster ophthalmicus (HZO) and the incidence of neovascular AMD. Indeed, because of a reactivation of VZV, HZO can be associated with ocular complications which are mostly located in the anterior segment of the eye and, more rarely, in the posterior segment.^81^ Comparing 1148 subjects aged >40 with HZO and 5740 controls matched by propensity score (including age, sex, socioeconomic factors, and some comorbidities), the authors observed an increased risk of neovascular AMD among HZO subjects (aHR = 4.62 95%CI [2.59–8.24], *P* < 0.001). Of note, this study suffers from the same limitations as those previously described for other studies using this database.

Regarding the suspected underlying mechanisms, VZV can infect the retina as shown by cases of necrotizing retinopathy which occur in severely immunosuppressed patients with HZO.^82^ To our knowledge, there are no data on VZV infection in the retina outside the context of immunosuppression. The authors suggested that retinal VZV infection could lead to local chronic inflammation, vasculopathy,^83^ as well as deposits of amyloid-β, which is a known component of drusen. Indeed, increased amyloid concentrations and aggregation were seen in vitro after VZV infection^84^ and in vivo in the plasma of subjects with an acute clinically diagnosed zoster.^85^

#### Cytomegalovirus

Because of its well-described role in promoting angiogenesis (reviewed in Caposio et al^86^) and the existence of rare cases reporting ocular neovascularization during *Cytomegalovirus* (CMV) retinitis,^87^ the potential involvement of CMV has been investigated in neovascular AMD.

An American cross-sectional study (previously described in the *C. pneumoniae* section)^36^ compared the titers of plasma anti-CMV IgG between 47 subjects with neovascular AMD, 36 with nonexudative AMD, and 67 controls. Although no data on the proportion of CMV-positive subjects was presented, univariate analyses highlighted that subjects with neovascular AMD had higher levels of anti-CMV IgG than both controls (*P* = 0.02) and subjects with nonexudative AMD (*P* = 0.06). The authors hypothesize that higher levels of anti-CMV IgG indicate a recent reactivation of the virus or a greater “total body burden of chronic CMV infection.” Conversely, there was no difference between subjects with nonexudative AMD and controls (*P* = 0.83). It should be noted that, although not statistically significant, the control group tended to be younger than the 2 AMD groups (71.6 ± 10.2 vs. 77.2 ± 7.39 in the nonexudative AMD group and 79 ± 6.49 in the neovascular AMD group; *P* = 0.24), suggesting a potential residual confounding bias.

Regarding the underlying mechanisms, several hypotheses have been made.

- Firstly, because monocytes are a major site of CMV latency, the virus can easily disseminate through the body and infiltrate various tissues. In the eye, CMV-infected monocytes become tissue-resident macrophages, cells which are suspected to have a direct role on the onset of AMD.^88^^,^^89^ Using C57BL/6 mice intraperitoneally infected with murine CMV, Cousins et al^89^ highlighted larger laser-induced CNV lesions in infected mice compared to controls. Their results suggested that this was not due to direct infection of choroidal tissues by murine CMV but rather related to the activation of CMV-infected macrophages towards a proangiogenic M2 phenotype.

- Secondly, infected macrophages can help to disseminate the virus to other types of cells within the eye. Among various types of mice intraperitoneally infected with murine CMV shortly after birth (i.e., with a not completely mature immune system that may favor virus dissemination),^90^, ^91^, ^92^ an American team highlighted that murine CMV establishes latent infection within the choroid and in a few RPE cells^90^^,^^91^ and reactivates following systemic immunosuppression.^91^ Latent ocular infection was also associated with increased levels of inflammatory and angiogenic factors (including IL-6 and VEGF)^90^^,^^91^ as well as retinal and choroidal pathologies (including deposits at basal and apical aspects of the RPE, photoreceptor degeneration, and CNV).^90^, ^91^, ^92^ To note, the number of macrophages observed in CNV lesions were similar in infected and uninfected VEGF-A^hyper^ mice and did not represent the majority of VEGF-positive cells.^90^ Subsequently, the authors also investigated the presence of CMV within human ocular tissues. Using droplet digital PCR assay performed on fresh eyes from 24 human cadavers (none of them having a known history of AMD), Xu et al highlighted that CMV DNA was detected in posterior eye cups (choroid/RPE) of 4 individuals (16.7%).^90^ Conversely, CMV DNA was not found in any of the anterior segment samples and very low DNA copy numbers were detected in the neural retina of a fifth individual (4.2%). Although the precise type of cells infected remains to be determined, these results led them to suggest that the choroid/RPE could be a fairly common site of latency for CMV in humans.

- Finally, a nonspecific impact of CMV on AMD has also been proposed,^36^ suggesting that infections by various pathogens (grouped under the concept of "total pathogen burden") could shape the immune response and favor the onset of AMD. Indeed, a study by Zinkernagel et al,^93^ using BALB/c mice intraperitoneally infected with murine CMV, showed that systemic murine CMV infection also led to an increased number of activated microglial cells within the subretinal space (located between the photoreceptors and the RPE and normally devoid of microglia), indicating low-grade inflammation implicated in AMD.

#### Human Herpes Virus 6A

Fierz et al^94^ published a hypothesis paper discussing the role of human herpes virus 6A (HHV-6A) in the onset of GA, mainly because of its known interaction with CD46, a membrane-bound inhibitor of complement activation. Indeed, serving as an HHV-6A receptor to enter cells, CD46 is downregulated following infection with HHV-6A. Thus, Fierz et al hypothesized that HHV-6A infection could explain the downregulation of CD46 seen in RPE cells at very early stages of GA^95^ and its potential influence on disease progression as CD46 knockout mice have been shown to spontaneously develop nonexudative AMD.^96^

#### Other Types of Studies

Four studies investigated the hypothesis of the involvement of infectious agents in the occurrence of AMD without focusing on a particular infectious agent.

- Using the American Framingham Heart and Eye Studies, an exploratory analysis published in 1977 evaluated potential risk factors for AMD^97^ and highlighted an increased risk of developing a “senile macular degeneration” for participants with a history of lung infections (analyses stratified by age categories and sex; no details on the pathogen involved) (As this study precedes the publication of all current widely used classification systems of AMD, we have refrained from harmonizing the term “senile macular degeneration.” According to the authors “senile macular degeneration” was positive when diagnosed by the definitive examiner together with best corrected central visual acuity of 20/30 or worse in the same eye).

- More recently, in 2016, aiming to explore interactions between genetic and environmental factors, Hoh Kam et al^98^ exposed CFH knock out mice to a pathogen free environment or to a conventional open environment during 9 months and investigated the effects on the retina. Compared with mice kept in a pathogen free environment, those in a conventional open environment presented (1) a higher number of macrophages in the outer retina and (2) higher levels of proinflammatory cytokines (TNF-α and IL-1β) in the outer retina and of stress markers (glial fibrillary acidic protein and vimentin) in the inner retina. Consistently, they also presented a reduced number of photoreceptors in the outer nuclear layer. Moreover, the authors stated that the results obtained with these CFH knock out mice were not found with wild-type mice (data not shown in the article). The authors conclude that, in genetically predisposed immunocompromised mice, environmental factors may trigger chronic inflammation in the retina and subsequent loss of photoreceptors.

- In 2015, Brosig et al^99^ stimulated cultured human RPE cells with analogs of (1) viral double-stranded RNA (dsRNA) (an intermediate of virus replication) and (2) viral/bacterial DNA to explore their effects on gene expression. Viral dsRNA induced the expression of the angiogenic factor basic fibroblast growth factor (but not of VEGF), of some inflammatory cytokines (IL-1β, IL-6, TNFα, monocyte chemoattractant protein-1, and macrophage inflammatory protein-2) and of complement proteins (complement factor B—the main activator of the alternative complement pathway—and to a lesser extent C5, C9, CFH but not C3). Conversely, viral/bacterial DNA had a lesser impact on gene expression, limited to an upregulation of some complement proteins (C5, C9). In view of these results, although the authors point out that differences in the concentrations used for viral RNA versus viral/bacterial DNA may partially explain the differences found, they concluded that alterations of RPE cells may be induced by viral dsRNA rather than viral/bacterial DNA.

- In 2021, a Chinese study investigated the presence of intraocular microbiota in the aqueous humor of patients undergoing cataract surgery with either AMD (n = 20), glaucoma (n = 26) or without other ocular disease (n = 41) using metagenomic sequencing.^100^ They highlighted the existence of a low-biomass intraocular microbiota. Moreover, although the intraocular microbiota of all patients and controls had in common to be mainly composed of bacteria, the 3 groups had significantly different alpha-diversities (*P* < 0.05). They also reported specific microbial signatures characterizing the intraocular environment of patients with AMD and glaucoma, suggesting that bacterial translocation may be associated with each condition. Regarding the underlying mechanisms, the bacterial translocation and also in general the confirmation of bacteria in the eye could be linked with the concept of ocular dysbiosis, which might favor certain eye diseases. However, it remains unclear if aging and disease lead to translocation of certain microbiota or if ocular microbiota exists since birth and only its composition might change due to disease.

### Brief Overview of the Literature on the Oral and Gut Microbiota

#### Oral Microbiota

Previously, 2 literature reviews^24^^,^^27^ investigated potential links between oral microbiota and AMD, with a particular focus on periodontal disease. Periodontal disease (and its more serious form called periodontitis) is an inflammatory disease triggered by several bacteria in dental plaque.

Briefly, 6 human studies with variable methodologies^101^, ^102^, ^103^, ^104^, ^105^, ^106^ investigated potential associations between various symptoms related to periodontal disease (fewer teeth, alveolar bone loss, presence of periodontal pockets, clinical attachment level, gingival index, periodontitis, etc) and AMD. Although a small case-control study found no associations between these symptoms and AMD,^101^ 4 cross-sectional studies with larger sample sizes,^102^, ^103^, ^104^, ^105^ highlighted a greater frequency of these symptoms among AMD cases compared to controls. Notably, in 2 of these studies,^104^^,^^105^ significant associations were restricted to a subgroup of participants <60 years of age. Moreover, one population-based cohort study based on a Health Insurance database^106^ showed an increased risk to develop AMD among subjects with prevalent periodontitis. Karesvuo et al^102^ also investigated the salivary presence of 6 pathogens (*Aggregatibacter actinomycetemcomitans, Porphyromonas gingivalis (P. gingivalis), Tannerella forsythia, Campylobacter rectus, Prevotella intermedia*, and *Treponema denticola*) in 54 self-reported AMD cases and 1697 controls but failed to find any difference in prevalence of bacterial DNA in the saliva between the 2 groups. More recently, a Canadian study^52^ (previously described in the paragraph on nasal microbiota) highlighted that 13 oral bacterial communities had an increased relative abundance in neovascular AMD cases (n = 13) compared to controls (n = 5), using 16s ribosomal RNA sequencing. Among these, the authors cited the *Rothia* genus (13-fold higher; *P* = 3.63E-18), the *Propionibacteriales* family (eightfold higher; *P* = 6.74E-09), the *Staphylococcus* species (sevenfold higher; *P* = 6.96E-05), and the *Cornyebacteriaceae* genus (sevenfold higher; *P* = 2.33E-05). Only the *Fusobacterium* genus (10-fold lower; *P* = 1.00E-10) and the *Bacilli* class (threefold lower; *P* = 0.007) had a decreased relative abundance in cases compared to controls.

Regarding the underlying mechanisms, the authors suggested that, secondary to periodontal disease, local inflammation, and ulceration of the periodontal epithelium could facilitate the systemic spread of either periodontal pathogens, microbial toxins (such as LPS), proinflammatory molecules, or immune complexes to distant sites such as the eye,^24^ favoring the onset of AMD.

Using in vivo and in vitro experiments, Arjunan et al investigated the potential impact of *P. gingivalis* (one of the major periodontopathogenic bacteria) on AMD. They highlighted that *P. gingivalis* can enter ARPE-19 cells and significantly reduce the expression of autophagy related genes.^107^ Subsequently, using C57BL/6 mice orally infected with *P. gingivalis*^108^ with and without a polybacterial suspension (referred as biofilm), they observed an increased size of laser-induced CNV lesions, a reduced retinal thickness and the presence of inflammatory drusen-like lesions in infected mice. Using quantitative PCR, they also confirmed the presence of *P. gingivalis* as well as an upregulated expression of angiogenic, proinflammatory, immunosuppressive, and oxidative stress genes and a downregulated expression of anti-inflammatory and antioxidative genes in the retina of infected mice, which, together, may promote the development of AMD.

#### Gut Microbiota

In recent years, several literature reviews have been published on the link between gut microbiota and AMD. Thus, the objective here is not to provide an exhaustive discussion of all original articles but, rather, to provide a brief overview and recent and relevant references to which the reader can refer.

We have identified 5 studies reporting original data on the link between gut microbiota and AMD in humans. Of all, the gut microbial profile was analyzed using DNA from fecal samples. However, it has to be noted that the information obtained from fecal samples does not represent the complete picture within the gut.^109^ Still, the available studies have revealed that specific bacteria are differentially abundant in patients with advanced AMD compared with healthy age-matched controls.^110^, ^111^, ^112^, ^113^, ^114^

- In 2017, using metagenomic sequencing, a Swiss study by Zinkernagel et al^110^ observed an increased *Firmicutes/Bacteroidetes* ratio in patients with neovascular AMD (n = 12), compared with healthy controls (n = 11). They also highlighted an enrichment in genera *Anaerotruncus, Oscillibacter* and in species *Ruminococcus torques* and *Eubacterium ventriosum* as well as a decreased abundance of species *Bacteroides eggerthii*.^110^

- In 2020, also using metagenomic sequencing, another study by the same Swiss study team (Zysset-Burri et al) showed that patients with neovascular AMD (n = 57) were enriched in class *Negativicutes* and less abundant in genus *Oscillibacter* and species *Bacteroides* than controls (n = 58).^111^

- A recent review published in 2021 mentioned unpublished data describing an enhanced abundance of the genera *Prevotella, Holdemanella*, and *Desulfovibrio* but reduced abundance of the genera *Oscillospira*, *Blautia*, and *Dorea* in patients with advanced AMD (n = 85) compared with healthy control subjects (n = 49).^112^

- In 2023, using 16s RNA sequencing, a case-control study from China^114^ reported increased abundances of *Bacteroides* and *Eubacterium_siraeum_group* genera and decreased abundances of *Blautia* and *Anaerostipes* genera in patients with advanced AMD (n = 30) compared with healthy controls (n = 17).

- In 2023, another case-control study from China using metagenomic sequencing^113^ observed a decreased *Firmicutes/Bacteroidetes* ratio in patients with neovascular AMD (n = 30) compared to controls (n = 30). They also observed an enrichment in genera *Veillonella* and *Lactobacillus* in patients with neovascular AMD, whereas the genera *Faecalibacterium*, *Anaerostipes*, *Blautia*, and *Eggerthella* were reduced. Furthermore, no difference regarding eukaryotic viruses was detected between patients with neovascular AMD and controls, whereas 50 phages were enriched and 62 phages depleted in patients with AMD. In order to identify common characteristics of AMD-associated gut microbial signatures in cohorts of different geographical origin, the authors integrated the publicly available datasets of the Swiss studies of Zinkernagel et al^110^ and Zysset-Burri et al.^111^ They highlighted that the compositions of the gut microbiome differed significantly between the Chinese and Swiss data (with approximately 75% bacterial species with different abundance). In the combined analyses, 3 bacterial species (*Ruminococcus callidus, Lactobacillus gasseri,* and an unknown species-level genome bin from the *Prevotellaceae* family) were concordantly enriched, and 6 bacterial species (*Cloacibacillus evryensis*, *Slackia piriformis*, as well as unknown species-level genome bins from the *Alistipes* and *Bilophila* genera and the *Bacteroidaceae* and *Acidaminococcaceae* family*)* depleted in AMD. Furthermore, the analyses showed that a total of 12 phages were enriched in AMD and 41 phages were reduced.

All in all, if a distinct microbial signature linked to AMD exists, the reported discrepancies highlighted in these studies make it difficult to link specific shifts in microbial composition to AMD.^25^^,^^115^, ^116^, ^117^

Several studies (in humans, animals, or in vitro) are also exploring the mechanisms potentially involved. Readers can refer in particular to the recent reviews by Zysset-Burri et al and Xiao et al^25^^,^^111^ for a more comprehensive discussion. In a nutshell, gut dysbiosis may impair the integrity of intestinal barrier allowing pathogens, bacterial endotoxins like LPS,^118^, ^119^, ^120^, ^121^ cytokines (among others IL-6, IL-1β, TNF-α, and VEGF-A), and other proinflammatory substances to pass into circulation, resulting in systemic inflammation, potential impairment of the immune response, and/or local ocular inflammation. Especially among subjects carrying the risk variant of the CFH gene, this could favor the development or progression of AMD.^111^ Both reviews also discuss the impact of diet (in particular high glycemic and high fat diets) on gut microbiota suggesting that gut microbiota may be part of the associations found between diet and AMD. Some authors also suggested that the impact of gut dysbiosis on AMD might be mediated by changes related to their metabolic functions or to the bioavailability of some nutrients (e.g., zinc or carotenoid).^26^

## Discussion

This systematic literature review gives an overview of studies investigating a potential link between infectious agents and AMD. It highlights that, although an overall low number of studies have been published on the subject so far (only 40 original articles excluding studies focusing on oral and gut microbiota), this research topic has been of growing interest in recent years. Of the 40 studies examined in this review, only 1 predates 2000, 12 were published between 2000 and 2010 (mainly on *C. pneumoniae*), followed by a substantial increase to 27 studies post 2010 (to which we can add the majority of studies on oral and gut microbiota).

Regarding the type of pathogens involved, a wide range of infectious agents has been investigated, including various microbiota (nasal, pharyngeal, oral, and gut microbiota), 8 bacteria (*C. pneumoniae, C. trachomatis, C. psittaci, E. coli, H. pylori, M. pneumoniae, M. chelonae,* and *B. megaterium*), 6 viral species (HIV, HBV, HCV, VZV, CMV, and HHV-6A), and 1 yeast (*C. albicans*). Among them, most have been investigated anecdotally (only 1 or 2 related studies) or only to test the specificity of the association for another pathogen (e.g., infections with different types of *Chlamydia*). Only *C. pneumoniae*, CMV, and HBV received more attention, with 17, 6, and 4 studies, respectively. It should be noted that, although 4 studies exist for HCV, HCV was not the main focus of 3 of them.

**Figure 2:**
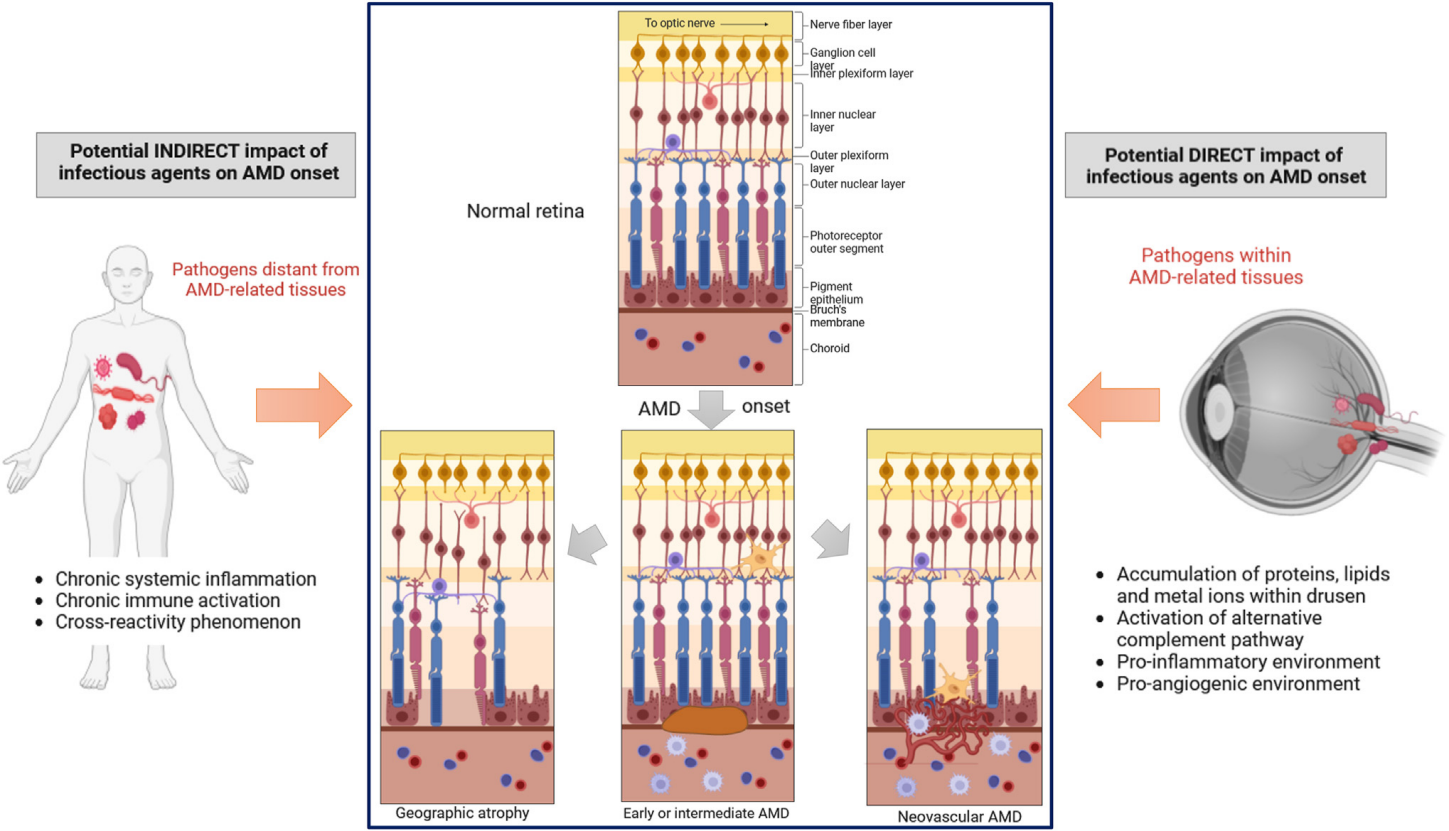
Stages of age-related macular degeneration (AMD) and potential involvement of infectious agents. Age-related macular degeneration is clinically classified into 3 stages according to the severity of fundus lesions. Early and intermediate AMD are described by the onset of drusen underneath the RPE and accumulation of immune cells (subretinal microglia and choroidal macrophages). Advanced AMD includes geographic atrophy, retinal thinning due to loss of photoreceptors, RPE cells, and choroidal capillaries, and neovascular AMD, invasion of new abnormal choroidal blood vessels and accompanying macrophages into the subretinal and/or sub-RPE space. Both geographic atrophy and neovascular AMD can also coexist in 1 eye (which is not shown in this figure). Several studies suggest a potential involvement of infectious agents in the development of AMD-related lesions: An indirect role of infectious agents has been suggested (i.e., a role of infections located distant from the eye), mainly related to their interactions with the immune system. Also, a direct role of some infectious agents has been discussed implying potential infection of various cells types within AMD-related tissues (e.g., RPE cells, endothelial cells, macrophages, retinal microglia). RPE = retinal pigment epithelium. Figure created with BioRender.com.

The numerous pathophysiological mechanisms investigated underline the diversity of interactions between infectious agents and AMD players (Fig 2):

- On the one hand, an indirect role of infectious agents in the onset of AMD has been suggested (i.e., a role of infections located distant from the eye), mainly related to their interactions with the immune system. Some authors proposed that chronic exposure to pathogens (or to their products such as bacterial endotoxin or soluble fungal ligands) could prime or partly activate immune cells located at a distance from the eye, leading to proinflammatory signaling and activating further inflammatory pathways or effector functions.^122^ Subsequently, such chronic systemic inflammation and/or the recruitment of immune cells within the eye could favor the onset of AMD. Thus, this hypothesis does not imply a particular pathogen but, rather, suggests an unspecific role of various infectious agents using a common mechanism (e.g., gut microbiota, oral microbiota, nasal or pharyngeal microbiota, *C. pneumoniae*, HIV, etc). In parallel, mechanisms more specific to a particular infectious agent (again at a distance from the eye) have also been proposed, including cross-reactivity phenomena because of sequence homologies between retinal and infectious proteins (e.g., HBV DNA polymerase and retinal S-Ag) leading to retinal damage.

- On the other hand, a direct role of infectious agents has been discussed implying potential infection of various cells types within AMD-related tissues (e.g., RPE cells, endothelial cells, macrophages, retinal microglia). Although only a few studies have investigated the presence of pathogens within AMD-related tissues in humans (4 studies on *C. pneumoniae*, 1 study on CMV, and 1 on *B. megaterium*), several mechanistic avenues have been considered in experimental studies. The ability of *C. pneumoniae* and CMV to infect monocytes, their potential secondary dissemination to AMD-related tissues during macrophage recruitment, and the potential to locally infect other cell types (e.g., endothelial cells for which *C. pneumoniae* has a particular tropism, RPE cells, which are the first line of defense against external pathogens, etc) have been most extensively studied. Some authors also suggest that the presence of dysbiosis in various microbiota (intestinal, oral, etc) could favor the translocation of pathogens from the bloodstream to ocular structures. Presence of infectious agents within AMD-related tissues have thus been associated with a proinflammatory and proangiogenic environment, which could favor AMD onset, in particular neovascular AMD. A role for potential infection of RPE cells by HHV-6A or HBV has also been suggested at other AMD stages like GA. Moreover, numerous other mechanisms implicated in AMD could also suggest an involvement of infectious agents: oxidative stress and the inflammatory component and in particular the activation of the alternative complement pathway whose main trigger is microbial in nature but also mechanisms such as mitochondrial and autophagy dysfunctions,^123^ accumulation within drusen^1^^,^^124^ of lipids,^125^ amyloid peptides,^126^ zinc^127^ and iron,^128^ which could be implicated in antimicrobial defense.

- Finally, certain risk or protective factors associated with AMD^1^ could also have a link with infections: (1) advancing age leading to the progressive onset of an immunosenescence and subsequent poorer infection control, (2) exposure to light which is a risk factor for certain infections (e.g. reactivation of certain herpes viruses),^129^ and (3) carotenoid and zinc supplementation, which could have an impact on antimicrobial defense.^127^^,^^130^^,^^131^

Despite these numerous hypotheses, the level of evidence remains low even for the 3 most studied pathogens:

- Briefly, results from human studies focused on *C. pneumoniae* are mixed, with 10 human studies in favor and 7 against its implication in AMD. Most of the negative studies were based on serological data, some of which suffering from methodological weaknesses (low numbers, cross-sectional or case-control design, little consideration of confounding, or interaction factors). To note, among the best quality studies, the one performed within the prospective “Cardiovascular Health and Age-related Maculopathy Study” presents a striking result suggesting a major increase in the risk of progression of early AMD in subjects with both high levels of anti *C. pneumoniae* IgG and the CC risk genotype of the CFH gene, a result that would need to be replicated. Two out of 3 studies also demonstrated the presence of *C. pneumoniae* within CNV lesions, suggesting a potential direct effect of the bacteria on AMD-related tissues. In vitro and animal studies also highlighted a proinflammatory and proangiogenic effect of *C. pneumoniae* infection, which could favor the onset of CNV.

- Regarding studies on HBV, an increased risk of AMD in infected subjects was demonstrated in 3 independent databases (2 South Korean cross-sectional studies and the Taiwanese Longitudinal Health Insurance Database 2000 which was the subject of 2 articles). The replicability of these results is intriguing and warrants further investigation of this hypothesis, particularly in countries with high endemicity of HBV infection.^132^ Furthermore, several underlying mechanisms have been suggested either through a cross-reactivity phenomenon due to sequence homology between retinal and viral components or through a sensitization of HBV-infected RPE cells to UV or blue light-induced cell death through downregulation of DNA repair pathways (although, to our knowledge, evidence of RPE cell infection by HBV in humans remains to be established).

- Regarding CMV, one postmortem study highlighted that CMV DNA was detected in posterior eye cups (choroid/RPE) of 16.7% of human cadavers, suggesting that the choroid/RPE could be a fairly common site of latency for CMV. Nevertheless, its impact on AMD remains poorly studied in humans with only 1 small cross-sectional study highlighting an association between high anti CMV IgG levels and neovascular AMD. Experimental studies are more numerous and suggest an impact of CMV on AMD onset through proangiogenic and proinflammatory mechanisms.

Our scoping review has some limitations. Given the diversity of terms associated with infectious agents, we had to limit our review to a single electronic database (PubMed) and search for terms only in titles and abstracts to ensure the feasibility of our work. Consequently, as mentioned above, we may have missed some articles studying the impact of infectious agents on the eye but without a specific focus on AMD (and therefore without AMD-related terms in the title and/or abstract). This was particularly the case for current studies focusing on SARS-COV2, in which SARS-COV2 infection related changes were reported on ocular structures including the retina. However, the context in which existing studies were set up is a limiting factor for investigating the impact of SARS-COV2 on the development of AMD (short follow-up time, scarcity of uninfected controls, absence of information on the presence/severity of AMD lesions before infection, etc). Furthermore, our search algorithm was not designed to identify studies investigating only certain components of pathogens (such as certain endotoxins, for example). Finally, our review was limited to articles in English and, as a result, 2 articles in Russian dealing with our research theme could not be included.

In conclusion, the currently available data do not clearly speak in favor or against the implication of infectious agents in AMD. Future studies combining human, animal and in vitro experiments for the same pathogen are needed to improve our understanding. Experimental studies will be decisive to decipher potential underlying mechanisms and could help to guide the choice of AMD stage to investigate for each suspected pathogen. Studying the interactions between an aging immune system and infectious agents also seems essential (i.e., could the onset of immunosenescence with advancing age explain a certain latency between the time of infection and the appearance of lesions?). Further anatomopathological studies assessing the presence or absence of pathogens within human AMD-related tissues will also help clarify the plausibility of a direct role for infectious agents. Large-scale epidemiological studies are needed to better take into account confusion bias and potential gene-environment interactions (i.e., is infection by a pathogen associated with the occurrence of AMD lesions only in genetically predisposed subjects?). Overall, an intensification of research efforts on the infectious hypothesis and AMD seems essential, given the potential repercussions in terms of diagnosis, prevention, and treatment.
