# Supplementary material for: Could Infectious Agents Play a Role in the Onset of Age-related Macular Degeneration? A Scoping Review
Source: Ophthalmol Sci. 2024 Nov 30;5(2):100668. doi: 10.1016/j.xops.2024.100668 (PMC11791433; doi:10.1016/j.xops.2024.100668)
Supplement: Appendix 1 [file mmc1.pdf]

Appendix 1: Search algorithm used in our systematic literature review

The following search algorithm was used in the Pubmed database:

((((((“Macular degeneration*”[Title/Abstract]) OR “age-related maculopath*”[Title/Abstract]) OR “age-related macular dystroph*”[Title/Abstract]) OR drusen* [Title/Abstract]) OR “geographic atrophy*”[Title/Abstract]) OR “choroidal neovascularization*” [Title/Abstract])

AND ((infect*[Title/Abstract]) OR bacteria*[Title/Abstract]) OR bacillus[Title/Abstract]) OR fungal[Title/Abstract]) OR fungus[Title/Abstract]) OR fungi[Title/Abstract]) OR mycos*[Title/Abstract]) OR parasite*[Title/Abstract]) OR virus[Title/Abstract]) OR viruses[Title/Abstract]) OR viral[Title/Abstract]) OR microbiotas[Title/Abstract]) OR microbiota[Title/Abstract]) OR microbial[Title/Abstract]) OR microbiome[Title/Abstract]) OR microbiomes[Title/Abstract]) OR microbes[Title/Abstract]) OR microbe[Title/Abstract]) OR flora[Title/Abstract]) OR microflora[Title/Abstract]) OR microorganism[Title/Abstract]) OR microorganisms[Title/Abstract]) OR “micro-organism”[Title/Abstract]) OR “micro-organisms”[Title/Abstract]) OR pathogen[Title/Abstract]) OR pathogens[Title/Abstract]) OR prion[Title/Abstract]) OR prions[Title/Abstract]) OR “anti-infective*”[Title/Abstract]) OR “anti infective*”[Title/Abstract]) OR antiinfective*[Title/Abstract]) OR antimicrobial*[Title/Abstract]) OR “anti-microbial*”[Title/Abstract]) OR “anti-bacterial*”[Title/Abstract]) OR antibacterial*[Title/Abstract]) OR “anti bacterial*”[Title/Abstract]) OR bactericidal[Title/Abstract]) OR bacteriocide*[Title/Abstract]) OR antibiotic[Title/Abstract]) OR antibiotics[Title/Abstract]) OR “anti-biotic”[Title/Abstract]) OR “anti-biotics”[Title/Abstract]) OR antifungal*[Title/Abstract]) OR “anti-fungal*”[Title/Abstract]) OR fungicide*[Title/Abstract]) OR antiparasitic*[Title/Abstract]) OR “anti-parasitic*”[Title/Abstract]) OR parasiticide*[Title/Abstract]) OR antiviral*[Title/Abstract]) OR “anti-viral*”[Title/Abstract]) OR “anti viral*”[Title/Abstract]) OR “anti-retroviral*”[Title/Abstract]) OR antiretroviral*[Title/Abstract]) OR “anti retroviral*”[Title/Abstract])

NOT (adeno-associated[Title/Abstract])
