# Supplementary material for: Could Infectious Agents Play a Role in the Onset of Age-related Macular Degeneration? A Scoping Review
Source: Ophthalmol Sci. 2024 Nov 30;5(2):100668. doi: 10.1016/j.xops.2024.100668 (PMC11791433; doi:10.1016/j.xops.2024.100668)
Supplement: Table S1 [file mmc2.pdf]

Table S1: Articles included in the systematic review

N°	First Author	Year	Infection agents	Type of study
1	Kalayoglu [33]	2003	*C. pneumoniae, C. trachomatis, E. Coli*	Human
2	Ishida [35]	2003	*C. pneumoniae*	Human
3	Miller [36]	2004	*C. pneumoniae, H. Pylori,* Cytomegalovirus	Human
4	Klein [31]	2005	*C. pneumoniae*	Human
5	Robman [37]	2005	*C. pneumoniae*	Human
6	Kalayoglu [34]	2005	*C. pneumoniae*	Human, *In vitro*
7	Kessler [39]	2006	*C. pneumoniae*	Human
8	Robman [38]	2007	*C. pneumoniae*	Human
9	Baird [40]	2008	*C. pneumoniae*	Human
10	Haas [41]	2009	*C. pneumoniae*	Human
11	Shen [47]	2009	*C. pneumoniae*	Human
12	Turgut [42]	2010	*C. pneumoniae, M. pneumoniae*	Human
13	Fujimoto [44]	2010	*C. pneumoniae*	Animal, *In vitro*
14	Khandhadia [46]	2012	*C. pneumoniae, C. trachomatis, C. psittaci*	Human
15	Wolf-Schnurrbusch [32]	2013	*C. pneumoniae*	Human
16	Nakata [43]	2015	*C. pneumoniae*	Human
17	Hata [45]	2023	*C. pneumoniae*	Animal, *In vitro*
18	Ho [51]	2018	Pharyngeal microbiota	Human
19	Rullo [52]	2020	Oral and nasal microbiota	Human
20	Wen [54]	2018	*B. megaterium*	Human
21	Collett [53]	2016	*M. chelonae*	Human
22	Maneu [55]	2014	*Candida albicans*	Animal
23	Jabs [56]	2015	Human immunodeficiency virus, Co-infection with Hepatitis C virus	Human
24	Jabs [57]	2017	Human immunodeficiency virus, Co-infection with Hepatitis C virus	Human
25	Roh [60]	2008	Hepatitis B virus, Hepatitis C virus	Human
26	Park [61]	2014	Hepatitis B virus	Human
27	Chou [62]	2018	Hepatitis B virus	Human, *In vitro*
28	Wu [63]	2019	Hepatitis B virus	Human
29	Yeh [76]	2021	Hepatitis C virus	Human
30	Ho [30]	2019	Varicella Zoster virus	Human
31	Cousins [89]	2012	Cytomegalovirus	Animal, *In vitro*
32	Zinkernagel [93]	2013	Cytomegalovirus	Animal

33	Xu [90]	2020	Cytomegalovirus	Human, Animal
34	Xu [91]	2021	Cytomegalovirus	Animal
35	Zhang [92]	2023	Cytomegalovirus	Animal
36	Fierz [94]	2017	HHV-6A	Hypothesis paper
37	Kahn [97]	1977	History of lung infections	Human
38	Hoh Kam [98]	2016	Pathogen free or conventional environment	Animal
39	Brosig [99]	2015	Viral RNA and viral/bacterial DNA	*In vitro*
40	Deng [100]	2021	Intraocular microbiota	Human
