# Supplementary material for: Could Infectious Agents Play a Role in the Onset of Age-related Macular Degeneration? A Scoping Review
Source: Ophthalmol Sci. 2024 Nov 30;5(2):100668. doi: 10.1016/j.xops.2024.100668 (PMC11791433; doi:10.1016/j.xops.2024.100668)
Supplement: Table S2 [file mmc3.pdf]

Table S2: Studies on bacteria and fungi included in the review - human studies then experimental studies

**First author**<br>**Year of publication**	**Kalayoglu et al.**<br>**2003**	**Ishida et al.**<br>**2003**	**Miller et al.**<br>**2004**
**Title**	*Serological association between Chlamydia pneumoniae infection and age-related macular degeneration*	*Is Chlamydia pneumoniae infection a risk factor for age related macular degeneration?*	*The Association of Prior Cytomegalovirus Infection With Neovascular Age-related Macular Degeneration*
**Journal**	Arch Ophthalmol	Br J Ophthalmol	Am J Ophthalmol
**Infectious agent investigated**	*> C. pneumoniae*<br>*> C. trachomatis*<br>*> E. coli*	*> C. pneumoniae*	*> C. pneumoniae*<br>*> H. pylori*<br>> Cytomegalovirus
**Original data source**<br>**Name**<br>+ (if available) location, type, baseline or period	> Country: USA<br>> Type: Case-control study including consecutive patients visiting Veterans Affairs (VA) Hospital Eye Clinic<br>> Baseline: 01.01.2001 -01.06.2001	> Country: Japan<br>> Type: Case-control study<br>> Baseline: no details	> Country: USA<br>> Type: Case-control study including patients examined at the Bascom Palmer Eye Institute or the Bascom Palmer Palm Beach Clinic<br>> Baseline: 10/2001 -12/2002
**Design and study sample**<br>**Design**<br>**Participants** (number + main inclusion and exclusion criteria)	> Design: Case-control study<br>> Participants:<br>*25 AMD<br>*18 controls<br><br>> Exclusion criteria: <55 years	> Design: Case-control study<br><br>> Participants:<br>*27 neovascular AMD<br>*22 age-matched controls<br><br>> Exclusion criteria: <60 or >79	> Design: Case-control study<br><br>> Participants:<br>*47 neovascular AMD<br>*36 dry AMD<br>*67 controls<br><br>>Exclusion criteria: age <50 or >90, HIV infection, malignancy, recent acute illness requiring hospitalization within 6 months, immunosuppressive condition
**Assessment of the infection**	> ELISA: Analyses of IgG antibodies to two *C. trachomatis* antigens (cHsp10 and cHsp60), two *E. coli* antigens (GroES and GroEL) and *C. pneumoniae* elementary bodies in serum.	> ELISA: Analyses of IgA and IgG antibodies to *C. pneumoniae* in serum.	> ELISA: Analyses of IgG antibodies to cytomegalovirus, *C. pneumoniae* and *H. pylori* in serum.
**Assessment of AMD**	> Classification was based on clinical examination.	> no details	> Classification was based on clinical examination, fundus photography and fluorescein angiography (using the International Classification for Age-related Macular Degeneration).
**Statistical methods**	> Univariate analysis: 2-tailed t-tests<br>> Multivariate regression was used to adjust for the variables independently associated with AMD: adjustment for age and smoking.	> Univariate analysis: Mann- Whitney test	> Univariate analysis: Student's t-test
**Results**	> In univariate analysis, subjects with AMD had increased titers of anti-Cp antibodies compared to controls (p=0.047).<br>> In multivariate analysis, this association remained stable (P=0.049).<br>> No association with AMD was found for *C. trachomatis* and *E. coli*.	> Subjects with AMD had increased IgG and IgA antibody titers against *C. pneumoniae* compared to controls (p = 0.006 for IgG; p = 0.005 for IgA).	> There was no significant difference in titers of IgG antibodies to *C. pneumniae* and *H. pylori* between neovascular AMD patients (p=0,35), dry AMD patients (p=0.80) compared to controls or between dry and neovascular AMD (p=0.55).

First author<br>Year of publication	Klein et al.<br>2005	Robman et al.<br>2005	Kalayoglu et al.<br>2005
**Title**	*Systemic Markers of Inflammation, Endothelial Dysfunction, and Age-Related Maculopathy*	*Exposure to Chlamydia pneumoniae Infection and Progression of Age-related Macular Degeneration*	*Identification of Chlamydia pneumoniae within human choroidal neovascular membranes secondary to age-related macular degeneration*
**Journal**	Am J Ophthalmol	Am J Epidemiol	Graefe's Arch Clin Exp Ophthalmol
**Infectious agent investigated**	> *C. pneumoniae*	> *C. pneumoniae*	> *C. pneumoniae*
**Original data source**<br>**Name**<br>+ (if available) location, type, baseline or period	Beaver Dam Eye Study cohort<br><br>> Country: USA<br>> Type: Prospective cohort<br>> Baseline: 01.03.1988 - 14.09.1990	Cardiovascular Health and Age-related Maculopathy Study<br>> Country: Australia<br>> Type: Participants with early AMD were chosen from two previous studies conducted in Melbourne, Australia: 1) the population-based Melbourne Visual Impairment Project (baseline: 1992–1994) and 2) the Vitamin E, Cataract, and Age-related Maculopathy Trial (VECAT) (baseline: 1995)<br>> Baseline: 1992 -1995	> Country: USA<br>> Type: Study analysing human tissue from the Massachusetts Eye and Ear Infirmary Eye pathology laboratory and the New England Eye Bank.<br>> Baseline: no details
**Design and study sample**<br>**Design**<br>**Participants** (number + main inclusion and exclusion criteria)	> Design :<br>Part 1: Nested case-control study<br>Part 2: Cohort study<br>> Participants:<br>Part 1: *188 early or late AMD<br>*195 controls matched for age, sex, and current smoking status<br>Part 2: 321 participants	> Design: Prospective study, assessment of progression in 2001/2002 using a six-level severity scale (with geography atrophy = level 5 and neovascular AMD = level 6)<br>> Participants:<br>*254 early AMD<br>> Exclusion criteria : age <50	> Design: Histopathological study<br>> Tissues:<br>*9 CNV due to AMD<br>*5 non-AMD CNV<br>*16 non-AMD eyes<br>All samples were formallin-fixed except 9 eyes from patients without AMD which were frozen.
**Assessment of the infection**	> Micro-immunofluorescence (MIF): Analyses of IgG antibodies to *C. pneumoniae* in serum.	> ELISA: Analyses of IgG antibodies to *C. pneumoniae* elementary body in serum.	> IHC: Staining with IgG antibody to *C. pneumoniae* in human tissue.<br>> PCR and sequencing: Detection and confirmation of *C. pneumoniae* DNA in human tissue.
**Assessment of AMD**	> Photographic macular grading followed the Wisconsin grading system.	> Photographic macular grading followed the grading system for AMD by the International ARM [Age-related Maculopathy] Epidemiological Study Group).<br>> Progression of AMD: Data were analyzed using two quantitative one qualitative definition of progression.	> no details
**Statistical methods**	> Part 1: Conditional logistic regression, stratified by age, sex, and smoking status.<br>> Part 2: Discrete linear logistic model, adjusted for age and sex. (Multivariate analysis controlling for age, sex, blood pressure, body mass index, lipid levels, history of gout, arthritis or emphysema, urinary tract infection, aspirin or corticosteroid use, smoking, heavy alcohol use, and multivitamin use.)	> Logistic regression models<br>> Full adjustments on age, smoking, family history of age-related macular degeneration, history of cardiovascular diseases, and source study	> no details
**Results**	> In part 1, there was no significant difference of seropositivy to *C. Pneumoniae* between AMD cases and controls (58% vs 61%, OR=0,90 [0,60-1,36], p=0.61), nor was there any difference when the analyses considered only cases of late AMD (OR=0,47 [0,19-1,16]), geographic atrophy (OR=0.77 [0,19-3,18]) or neovascular AMD (OR=0,35 [0,11-1,12]).<br><br>> In part 2, seropositivity to *C. pneumoniae* was not associated to either 10-year incidence of AMD or its 10-year progression.	> After multivariate adjustment, subjects in the 2nd tertile and in the 3rd tertile of anti-Cp IgG titers had significant increased risk of 7-year AMD progression (using the definition referred to as "fine grading" by the authors) compared to those in the 1st tertile (aOR=2.10 [1.00-4.50] and aOR=2.58 [1.24-5.41], respectively).<br><br>> Using two others definitions of AMD progression ("coarse grading" and "side-by-side grading"), results were broadly similar but not significant for the "coarse grading".	<u>> IHC:</u> 4 of 9 AMD- CNV specimens (44%) showed evidence of *C. pneumoniae* (0 of 7 non-AMD controls and 0 of 5 non-AMD CNVs).<br><u>> PCR and sequencing:</u> 2 of 9 AMD- CNV specimens (22%) showed evidence of *C. pneumoniae* (0 of 9 non-AMD controls).

First author<br>Year of publication	Kessler et al.<br>2006	Robman et al.<br>2007	Baird et al.<br>2008
**Title**	*Chlamydia pneumoniae is not detectable in subretinal neovascular membranes in the exudative stage of age-related macular degeneration*	*Exposure to Chlamydia pneumoniae Infection and Age-Related Macular Degeneration*	*Gene–environment interaction in progression of AMD: the CFH gene, smoking and exposure to chronic infection*
**Journal**	Acta Ophthalmol Scand	Invest Ophthalmol Vis Sci	Human Molecular Genetics
**Infectious agent investigated**	> *C. pneumoniae*	> *C. pneumoniae*	> *C. pneumoniae*
**Original data source**<br>**Name**<br>+ (if available) location, type, baseline or period	> Country: Germany<br>> Type: Study analysing human tissue collected from consecutive patients<br>> Baseline: 04/1998 - 10/1999	Blue Mountains Eye Study survey<br><br>> Country: Australia<br>> Type: Prospective cohort<br>> Baseline: 1992–1994	Cardiovascular Health and Age-related Maculopathy Study<br><br>> Country: Australia<br>> Type: Participants with early AMD were chosen from two previous studies conducted in Melbourne, Australia: 1) the population-based Melbourne Visual Impairment Project (baseline: 1992–1994) and 2) the Vitamin E, Cataract, and Age-related Maculopathy Trial (VECAT) (baseline: 1995)<br>> Baseline: 1992 -1995
**Design and study sample**<br>**Design**<br>**Participants** (number + main inclusion and exclusion criteria)	> Design: Histopathological study<br><br>> Frozen Tissues:<br>*13 CNV due to AMD	> Design: nested case–control sample<br><br>> Participants:<br>*197 AMD (159 early, 38 advanced)<br>*433 controls matched for age, sex and smoking status	> Design: Prospective assessment of progression in 2001/2002<br><br>> Participants:<br>*233 early AMD<br><br>>Inclusion criteria: availabilty of genotyping information<br>>Exclusion criteria: age <50
**Assessment of the infection**	> PCR: Detection of *C. pneumoniae* DNA<br>> Broad-range bacterial 16S rDNA PCR	> ELISA: Analyses of IgG antibodies to *C. pneumoniae* elementary body in plasma.	>ELISA: Analyses of IgG antibodies to *C. pneumoniae* elementary body in serum.
**Assessment of AMD**	> Classification was based on clinical examination and fluorescein angiography.	> Photographic macular grading followed the Wisconsin grading system.	> Photographic macular grading followed the grading system for AMD by the International ARM [Age-related Maculopathy] Epidemiological Study Group).<br>> Progression of AMD: Data were analyzed using two quantitative one qualitative definition of progression.
**Statistical methods**	> no details	> Logistic regression models, adjusted for age, sex and smoking status<br>> Full adjustment: Age, sex, smoking status, AMD family history, history of heart attack.	> Univariate analysis: $\chi 2$ test<br>> Multivariate logistic regression analysis: adjusted for age, smoking, follow-up time, sex and study source
**Results**	> *C. pneumoniae* DNA was not detected in any subretinal neovascular membranes.<br>> Notably, no DNA of other pathogens was identified using a broadrange bacterial 16S rDNA PCR.	> In multivariate analysis, titers of anti-*C. pneumoniae* IgG were neither associated with prevalent AMD (n=197 cases of AMD, aOR 1.02 [0.66–1.56] when comparing the 3rd tertile to the 1st tertile), nor when early (n=159 cases, aOR=0.97 [0.61-1.53]) or late AMD (n= only 38 cases, aOR=1.23 [0.52-2.92]) were analyzed separately. Full adjustement on family history of AMD and history of cardiovascular disease did not change the results.<br>> Similar analyzes were also performed on "incident" early and late AMD defined according to the evolution between the BMES I and II surveys. *C. pneumoniae* antibody titers was not associated with incident early (n=87, aOR=1.08 [0.68-1.99]) or late (n=22, aOR 1.85, 95% CI 0.57–6.05) AMD.	> -In multivariate models, while the CC genotype was associated with an increased risk of AMD progression (aOR=2.43 [1.07-5.49], p=0.03 compared to the TT genotype), having both the CC genotype and being within the upper tercile of antibodies to *C. pneumoniae* was associated with a greater risk (aOR=11.8 [2.1-65.8], p=0.005 compared to TT genotype within the lowest tertile).

First author<br>Year of publication	Haas et al.<br>2009	Shen et al.<br>2009	Turgut et al.<br>2010
**Title**	*Complement factor H gene polymorphisms and Chlamydia pneumoniae infection in age-related macular degeneration*	*Chlamydia pneumoniae infection, complement factor H variants and age-related macular degeneration*	*Mycoplasma Pneumoniae and Chlamydia Pneumoniae Seropositivity in Patients With Age-Related Macular Degeneration*
**Journal**	Eye	Br J Ophthalmol	J Clin Med Res
**Infectious agent investigated**	> *C. pneumoniae*	> *C. pneumoniae*	> *Mycoplasma Pneumoniae*<br>> *C. Pneumoniae*
**Original data source**<br>**Name**<br>+ (if available) location, type, baseline or period	> Country: Austria<br>> Type: Case-control study including patients examined at the Department of Ophthalmology at the Rudolf Foundation Hospital, Vienna<br>> Baseline: no details	National Eye Institute cohort<br><br>> Country: USA<br>> Type: Case-control study<br>> Baseline: no details	> Country: Turkey<br>> Type: Case-control study<br>> Baseline: no details
**Design and study sample**<br>**Design**<br>**Participants** (number + main inclusion and exclusion criteria)	> Design: Case–control study<br><br>> Participants:<br>*75 AMD<br>*75 controls<br>>Inclusion criteria: ≥ 55 years<br><br>>Exclusion criteria: hereditary diseases, polypoidal choroidal vasculopathy, secondary CNV due to myopia, angioid streaks, inflammatory or infectious chorioretinal disease, trauma, or diabetic retinopathy	> Design: Case-control study and histopathological study<br><br>> Participants with available peripheral blood cells:<br>*148 advanced AMD<br>*162 controls<br><br>> Participants with available paraffin-embedded ocular slides :<br>*59 late AMD (geographic atrophy or CNV)<br>*16 age-matched controls	> Design: Case-control study<br><br>> Participants:<br>*20 wet AMD<br>*20 dry AMD<br>*20 age and sex-matched controls<br><br>> Exclusion criteria: Diabetes mellitus, ocular or systemic infection and inflammation, hematological and immune disease, malignancy, hypergammaglobulinemia, connective tissue disease, history of myocardial infarction and coronary artery disease, history of usage of drugs influencing serum immunoglobulin levels, previous laser treatment, intravitreal injection
**Assessment of the infection**	> ELISA: Analyses of IgG antibodies to *C. pneumoniae* elementary body in serum.	> PCR: Amplification of the *C. pneumoniae* 16S rRNA gene of peripheral blood cells and macular cells of paraffin-embedded ocular slides.	> ELISA: Analyses of *C. pneumoniae* and *Mycoplasma pneumonia* IgM and IgG antibody titers in serum.
**Assessment of AMD**	> Classification was based on clinical examination, optical coherence tomography and fluorescein/indocyanine angiography.	> Classification was based on fundus photographs.	> Classification was based on clinical examination, fundus imaging, optical coherence tomography and fluorescein angiography.
**Statistical methods**	> Univariate analysis: t-test, χ2-test.<br>> Binary logistic regression analysis	> Univariate analysis: χ2-test<br>> χ2 tables were used to assess the associations between CFH variants, AMD and *C. pneumoniae* infection.	> Univariate analysis: Student's t-test, χ2-test
**Results**	> In univariate analysis, seropositiviy to *C. pneumoniae* did not differ between AMD patients and controls (p = 0.192).<br><br>Notably, the interaction between genetic factors and *C. pneumoniae* could not be assessed due to the small sample size.	> Blood cells: Chronic *C. pneumoniae* infection was more often exhibited in AMD patients (20.3% - n=30) than controls (10.5% - n=17) (p=0.017).<br><br>No significant interaction was found between infection with *C. pneumoniae* and CFH variant.<br><br>> Paraffin-embedded ocular slides: *C. pneumoniae* DNA was found in macular cells of 2 cases (3.3%) and 1 control (6.2%) (not statistically significant).	> No significant difference was found in the distribution of IgM and IgG antibody titers to *C. pneumoniae* and *M. pneumoniae* in patients with wet and dry AMD, and in controls (p > 0.05).<br>> The seroprevalences reported in this article were very low: 0%, 10% and 0% for *C. pneumoniae* and 10%, 5%, 5% for *M. pneumoniae* in patients with dry AMD, wet AMD and controls, respectively.

**First author**<br>**Year of publication**	**Khandhadia et al.**<br>**2012**	**Wolf-Schnurrbusch et al.**<br>**2013**	**Nakata et al.**<br>**2015**
**Title**	*Chlamydia infection status, genotype, and age-related macular degeneration*	*Detection of Chlamydia and Complement Factors in Neovascular Membranes of Patients with Age- related Macular Degeneration*	*Calcium, ARMS2 Genotype, and Chlamydia Pneumoniae Infection in Early Age-Related Macular Degeneration: a Multivariate Analysis from the Nagahama Study*
**Journal**	Molecular Vision	Ocular Immunology and Inflammation	Sci Rep
**Infectious agent investigated**	> *C. pneumoniae*<br>> *C. trachomatis*<br>> *C. psittaci*	> *C. pneumoniae*	> *C. pneumoniae*
**Original data source**<br>**Name**<br>+ (if available) location, type, baseline or period	> Country: UK<br>> Type: Case-control study<br>> Baseline: no details	> Country: Switzerland<br>> Type: Study analysing human tissue collected from the Inselspital, Berne, Switzerland.<br>> Baseline: January 1998 and December 2004	Nagahama Study<br>> Country: Japan<br>> Type: Community-based prospective cohort study<br>> Baseline: 11/2008 - 11/2010
**Design and study sample**<br>**Design**<br>**Participants** (number + main inclusion and exclusion criteria)	> Design: Case-control study<br>> Participants:<br>*199 AMD<br>*100 controls<br>> Inclusion criteria: ≥ 50 years, Caucasian origin, confirmed genotyping (Both cases and controls were selected based on CFH Y402H variant genotype status (1/3 homozygous CC, 1/3 heterozygous CT, 1/3 wild-type TT)	> Design: Histopathological study<br>> Paraffin-embedded tissues:<br>*26 CNV due to AMD<br>> Exclusion criteria: prior treatment of choroidal neovascularization (e.g., photodynamic therapy, focal laser coagulation, injections with steroids or anti-VEGF substances)	> Design: Cross-sectional survey<br>> Participants:<br>*971 large drusen cases (soft distinct and soft indistinct drusen ≥125 mm in diameter)<br>*3,209 controls<br>> Exclusion criteria: other retinal diseases that would affect grading of macular lesion<br>> Inclusion criteria: age ≥ 50 years
**Assessment of the infection**	> MIF: Analyses of IgG antibodies to *C. pneumoniae, C. trachomatis*, and *C. psittaci* IgG in plasma.	> IHC: Staining with IgG antibody to *C. pneumoniae*	> Immunological Assay (not specified): Analyses of IgA and IgG antibodies to *C. pneumoniae*<br>(To note, IgA were not included in the statistical models due to collinearity.)
**Assessment of AMD**	> Classification was based on clinical examination, stereoscopic fundus photographs, and fluorescein angiography, using the AgeRelated Eye Disease Study (AREDS) grading system.	> Classification was based on clinical examination, fundus photography and fluorescein angiography, according to the Macular Photocoagulation Study.	> Photographic macular grading was performed twice by two independent ophthalmologists according to the simplified severity scale for age-related macular degeneration in the Age-Related Eye Disease Study (AREDS).
**Statistical methods**	> Univariate analysis: χ2 test, Fisher Exact test, Student's t-test<br>> Logistic regression adjusted for *C. pneumoniae* seropositivity, age, sex, BMI, CFH Y402H genotype, HTRA1 genotype	> Univariate analysis: Two-way ANOVA with Fisher`s exact test	> Univariate analysis: ANOVA, χ2 test<br>> Multivariate logistic regression model adjusted for: blood pressure, BMI, high-density lipoprotein [HDL] cholesterol, high-sensitivity C-reactive protein [hs-CRP], smoking status and genetic factors
**Results**	> In multivariate logistic regression analysis, seropositivity to *C. pneumoniae* was not predictive of AMD status (p=0.768) or AMD severity (no p-value provided).<br>> In univariate analyses, seropositivity to *C. pneumoniae*, *C. trachomatis* or *C. psittaci* were not associated with AMD status (p=0.838, 0.268 and 0.504, respectively). Among AMD cases, C. trachomatis and C. psittaci were not associated with AMD severity (p=0.762 and 0.127) while the p-value for *C. pneumoniae* was nearly significant (p=0.057 with 58.5% of infected subjects among early AMD and 72.6% among late AMD).<br>> When analyses were restricted to i) carriers of CFH Y402 risk C allele and then ii) carriers of HTRA1 RS11200638 risk A allele, none of the three species were significantly associated with AMD status. Nevertheless, among carriers of CFH Y402 risk C allele, seropositivity for C pneumoniae differed from early and late AMD (p=0.091, 60% of infected subjects in early AMD vs. 75% in late AMD). Notably, among carriers of each genetic risk, significant associations between seropositivity to *C. psittaci* were found with less infected subjects among late AMD cases.	> Seventeen membranes (68%) were positive for *C. pneumoniae.*<br>The percentage of *Chlamydia*-positive cells varied from 1 to 18% (mean: 6 ± 3%).<br>Notably, macrophages were detected in 19 membranes (76%) including 16 positive to *C. pneumoniae.*<br><br>> CNV mean area was not statistically different between *C. pneumoniae* positive and negative samples.	> In univariate analyses, *C. pneumoniae* IgG levels were significantly increased in cases compared with controls (1.30 and 1.23, respectively; P<0.001) and *C. pneumoniae* infections was more frequent in cases (56.4%) compared with controls (51.7%; P=0.01).<br>> In multivariate analyses, *C. pneumoniae* IgG levels were significantly associated with drusen cases (aOR=1.020 [1.001 - 1.040], p<0.05).

First author<br>Year of publication	Collett et al.<br>2016	Ho et. Al<br>2018	Rullo et al.<br>2020	Deng et al.<br>2021
**Title**	*Effects of Disseminated Mycobacterial Infection on Age-Related Macular Degeneration*	*Human pharyngeal microbiota in age-related macular degeneration*	*Local oral and nasal microbiome diversity in age-related macular degeneration*	*Identification of an intraocular microbiota*
**Journal**	Case Rep Ophthalmol	PloS one	Scientific reports	Cell discovery
**Infectious agent**	> *Mycobacterium chelonae*	> Pharyngeal microbiota	> Oral and nasal microbiome	> Intraocular microbiota
**Original data source**<br>**Name**<br>+ (if available) location, type, baseline or period	> Country: USA<br>> Type: case report<br>> Baseline: no details	> Country: Singapur<br>> Type: Patients examined at at three clinical sites: Singapore National Eye Centre, Tan Tock Seng Hospital and National University Hospital<br>> Study Period: no details	> Country: Canada<br>> Type: no details<br>> Study Period: no details	> Country: China<br>> Type: Patients examined at Zhongshan Ophthalmic Center (China) and Tianjin Medical University Eye Hospital (China)<br>> Study Period: 09/2014 - 08/2018
**Design and study sample**<br>**Design**<br>**Participants** (number + main inclusion and exclusion criteria)	> Design: case report<br>> Participant: 1 with subacute bilateral transformation from nonexudative to exudative AMD associated to a bilateral choroiditis	> Design: case-control study<br>> Participants:<br>*245 AMD cases (80 early and 165 late)<br>*386 controls	> Design: case-control study<br>> Participants: 13 neovascular AMD , 5 controls<br>> Inclusion criteria: age > 50 years<br>> Exclusion criteria: Antibiotherapy, exclusive dry AMD. Controls were excluded if there was a past history of retinal co-morbidity.	> Design: Analysis of surgically obtained aqueous humor<br>> Participants: Patients undergoing cataract surgery including 20 AMD, 26 glaucoma, 41 controls without other ocular diease
**Assessment of the infection**	> Sputum cultures: Detection of *M. chelonae*<br>> PCR: Detection of *M. chelonae* in serum	> 16s rRNA sequencing using throat swabs<br>> In a subsample (20 cases, 20 controls), quantitative-PCR were then performed to validate the ability of 16s rRNA sequencing to detect differences in abundance for three highly prevalent genera.	> 16s rRNA sequencing using buccal and nasal mucosa swabs.	> Metagenomic sequencing.
**Assessment of AMD**	> Diagnosis was based on clinical examination and optical coherence tomography.	> Classification was based on clinical examination, optical coherence tomography, fluorescein angiography (graded using a modification from the Macular Photocoagulation Study), and ICG angiography (graded using the Japanese Study Group guidelines).	> Classification was based on clinical examination. Neovascular AMD was confirmed on spectral domain-optical coherence tomography and intravenous fluorescein angiography.	> no details
**Statistical methods**	n/a	> Comparison of Shannon's and Simpson's α diversity indices<br>> Principal component analyses of relative abundances at the genus level<br>> Differential abundance analysis at the genus level between case/control and early/late AMD were performed using i) the "DESeq2" package and ii) Generalised Linear Mixed Model with P-values corrected for multiple testing	> Characterization of distinct microbial community was performed using principal coordinates analysis of the Bray-Curtis index<br>> Comparative analysis between cases and controls was carried out with the DESeq2 package.	> Comparison of alpha-diversities (Shannon index and evenness)<br>> Principal Coordinates Analysis on the composition of the intraocular microbiota<br>> Hierarchical clustering analysis of the relative abundance of functional microbial genes
**Results**	> Mycobacterial dissemination may have induced bilateral choroiditis, triggering the transition from preexisting nonexudative AMD towards exudative AMD.	> α diversity indices did not differ between cases and controls<br>> There was a lack of clustering between AMD and controls in principal component analysis.<br>> Using DESeq2, compared to controls, the relative abundance of *Gemella* genus was increased among AMD cases while that of *Prevotella* genus was decreased. No differences were found between early and late AMD cases.<br>> Using Generalised Linear Mixed Model analysis, enriched *Gemella* (Adj-p = 0.007) and *Streptococcus* (Adj-p = 0.002) and reduced *Prevotella* (Adj-p = 6.95E−5) and *Leptotrichia* (Adj-p = 0.007) were associated with AMD cases and not controls.<br>> Stratifying the AMD cases by disease into early and late stages, *Prevotella* and *Leptotrichia* relative abundance was significantly lower in late AMD samples than controls . Conversely, late AMD samples were revealed to have greater relative abundances of *Streptococcus* and *Gemella* (*Streptococcus* Adj-p = 1.19 x 10−4; *Gemella* Adj-p = 0.028).<br>> Analysing the >60 years subset revealed only *Prevotella*, *Leptotrichia* and *Streptococcus* to be significantly associated (*Streptococcus* Adj-p = 1.85 x 10−5; *Leptotrichia* Adj-p = 0.005; *Streptococcus* Adj-p = 0.035) among cases and controls.	> Nasal streptococcus (P = 0.011), *Burkholderiales* order (P = 3.29E-05), *Actinomycetaceae* family (p = 3.73E-06), *Gemella* genus (p = 0.0002), *Proteobacteria* family (p = 0.004), *Actinomyces* species (p = 0.002), the *Streptococcus* species (P = 0.011), and *Veillonella* species (p = 0.005) were significantly higher in cases compared to controls. Members of the *Clostridia* class were 4-fold higher in controls as compared to cases (p = 0.007).<br>> In the oral mucosa, *Propionibacteriales* family (p = 6.74E-09), *Rothia* genus (p = 3.63E-18), *Staphylocococus species* (p = 6.96E-05) and *Cornyebacteriaceae* genus (p = 2.33E-05) were increased over controls, *Fusobacterium* genus (p = 1.00E-10) and *Bacilli* class (p = 0.007) were statistically higher in controls compared to cases.	> The three groups had significantly different alpha-diversities and could be differentiated by a microbial signature.

**First author**<br>**Year of publication**	**Kalayoglu et al.**<br>**2005**	**Fujimoto et al.**<br>**2010**	
**Title**	*Identification of Chlamydia pneumoniae within human choroidal neovascular membranes secondary to age-related macular degeneration*	*Choroidal Neovascularization Enhanced by Chlamydia pneumoniae via Toll-like Receptor 2 in the Retinal Pigment Epithelium*	
**Journal**	Graefe's Arch Clin Exp Ophthalmol	Invest Ophthalmol Vis Sci	
**Type**	In vitro	Animal	In vitro
**Infectious agents**	> *C. pneumoniae*	> *C. pneumoniae*	> *C. pneumoniae*<br>> LPS
**Cell line or animal models**<br>**Exposition**	> Cell line:<br>(i) Human monocyte-derived macrophages<br>(ii) Human RPE cells (ARPE-19)<br><br>> Exposition: infection with varying doses of *C. pneumoniae* or mock-infected	> Animal models:<br>(i) C57BL/6 mice<br>(ii) Knockout mice for either MyD88, TLR2 and TLR4<br><br>> Exposition:<br>1. Experimental CNV by laser photocoagulation (LP)<br>2. Vitreous injection of the *C. pneumoniae* antigen (control: PBS) immediately after LP	> Cell line:<br>(i) Primary-cultured RPE cells of C57BL/6 origin<br>(ii) Primary-cultured RPE cells of knockout mice for MyD88, TLR2 and TLR4<br><br>> Exposition:<br>Stimulation with *C. pneumoniae* antigen (1, 5 or 25 µg/ml), LPS (1 µg/ml) or unstimulated
**Main outcomes and results**	**(i) Quantification of pro-angiogenic cytokines (VEGF, IL-8 and MCP-1) by ELISA**:<br>> *C. pneumoniae* infected both types of cells.<br>> Infection with *C.pneumoniae* led to an increase in VEGF secretion by macrophages (in a time and dose-dependent manner) but not in RPE cells (which already secreted high basal levels of VEGF).<br>> Infection with *C. pneumoniae* led to an increase in IL-8 and MCP-1 secretion by RPE cells (in a time and dose-dependent manner).	**(i) Quantification of CNV area in choroidal flat mounts 7 days after LP:**<br>- Size of CNV was shown to be significantly larger in the *C. pneumoniae* antigen-inoculated wild type mice and TLR4 KO mice, but not TLR2 KO mice compared to PBS-inoculated wild type mice.<br>- Anti–TLR2 blocking mAb-treated wild type mice lost CNV enhancement by *C. pneumoniae* antigen injection, but not anti–TLR4 blocking mAb-treated wild type mice. Importantly, TLR2 agonists (without *C. pneumoniae*) can also increase CNV size.<br><br>**(ii) Detection of cytokines in the intraocular fluid of wild type mice by ELISA:** IL-6 and VEGF were significantly increased in *C. pneumoniae*-inoculated mice compared to PBS-inoculated mice.	**(i) Quantification of cytokines by real-time RT-PCR / ELISA in RPE cells:**<br>- *C. pneumoniae* antigen stimulation increased the mRNA expression of IL-6 in the supernatant of RPE cells of wild type mice in a dose-dependent manner compatible with LPS stimulation.<br>- *C. pneumoniae* antigen induced VEGF expression in RPE cells of wild type mice comparable to LPS stimulation, but not TNF-α expression.<br>- *C. pneumoniae* antigen mediated IL-6 and VEGF expression were significantly reduced in RPE cells from MyD88 KO and TLR2 KO mice, but not TLR4 KO mice, compared to wild type mice.<br><br>**(ii) Detection of TLR2 via real-time RT PCR +/- immunostaining in RPE cells of wild type and TLR2 KO mice:** TLR2 was expressed by unstimulated RPE cells. The intensity of TLR2 was augmented by LPS or *C. pneumoniae* antigen stimulation, and protein expression of TLR2 was confirmed in LPS-stimulated RPE cells by immunostaining.

First author<br>Year of publication	Hata et al.<br>2023		Maneu et al.<br>2014
**Title**	*Early-life peripheral infections reprogram retinal microglia and aggravate neovascular age-related macular degeneration in later life*		*Retinal Microglia Are Activated by Systemic Fungal Infection*
**Journal**	Journal of Clinical Investigation		Ophthalmol Vis Sci.
**Type**	Animal	In vitro	Animal
**Infectious agents**	> *C. pneumoniae*<br>> LPS	> *C. pneumoniae*<br>> LPS	> Candida albicans
**Cell line or animal models**<br>**Exposition**	> Animal models:<br>(i) C57BL/6J mice<br>(ii) heterozygous Cx3cr1$^{CreER/+}$:R26$^{iDTR/+}$ mice (to investigate the role of retina-resident myeloid cells)<br>(iii) homozygous B6.129S7-Rag1^{tm1Mom} /J (referred to as Rag1$^{-/-}$) mice (to investigate the role of B and T cells)<br>(iv) heterozygous Cx3cr1$^{GFP/+}$ mice<br><br>> Exposition:<br>1. Intraperitoneal inoculation of the C. pneumoniae and induction of CNV by laser photocoagulation (LP) 60 days post-infection<br>2. Intraperitonal LPS injection either mimicking an acute exposure (1 low dose injection of 0,5mg/kg) or a sustained infection (injections on 4 consecutive days) followed by induction of CNV by laser photocoagulation (LP) 4 weeks after LPS injection	> Cell lines:<br>(i) Bone marrow cells derived from LPS-pretreated mice and control mice were differentiated into mature macrophages and restimulated with LPS<br>(ii) Monocytes from the bone marrow of laser-burned mice, either preconditioned with LPS or with control PBS, were cocultured with choroidal explants.	> Animal model:<br>C57BL/6J mice<br><br>> Exposition: Intravenous administration of C. albicans
**Main outcomes and results**	**(i) Quantification of CNV area in choroidal flat mounts of mice 14 days after LP:**<br>- 50% increase in CNV area in C57BL/6J mice previously infected with C. pneumoniae compared with control mice. Notably, similar numbers of recruited mononuclear phagocytes were evidenced in infected and control mice.<br>- LPS heightens CNV formation and no difference were observed regarding the number of recruited mononuclear phagocytes.<br>- Sustained LPS exposure did not heighten CNV formation in mice with decreased retina-resident myeloid cells.<br>- Sustained LPS exposure heightens CNV formation in mice lacking B and T cells, supporting a mechanism independent of adaptive immunity.<br>**(ii) Quantification by RT-qPCR of innate immunity–related gene expression in retina/choroid complexes after LPS inoculation and laser-induced injury :** Il1b, Il6, Tgfb1, Tlr4, and Aif1 expression was decreased compared to control, suggesting an altered retinal immune response following LPS exposure. Increased expression of Vegfa was also highlighted in the choroid for the 4×LPS group.<br>**(iii) Identification of epigenetically altered subpopulations of CX3CR1+ myeloid cells by single-cell assay for transposase-accessible chromatin with high-throughput sequencing (scATAC-seq) and identification of cluster-specific epigenetic changes:** Prior sustained exposure to LPS leads to CX3CR1+ retinal microglia which are epigenetically distinct from microglia in retinas from naive mice and mice undergoing CNV without prior exposure to LPS. The gene of ATF3 (Activating transcription factor 3) was preferentially epigenetically modulate and its induction mitigated inflammatory response.	**(i) Bulk RNA sequencing of bone marrow-derived macrophages from control mice or LPS-pretreated mice which were restimulated with LPS:** Prior exposire to LPS induced transcriptional reprogramming toward reduced inflammatory but enhanced angiogenic phenotypes.<br>**(ii) Assessment of polarization of macrophages from LPS-preconditioned mice by flow cytometry:** significant increase in CD206- expressing M2-like cells and a decrease in M1-like cells (Rebalancing toward a M2-like state is linked to heightened angiogenesis.)<br>**(iii) Assessment of sprouting area in choroid explants:** Significant increase in sprouting area in explants cocultured with myeloid cells from LPS-primed mice compared to PBS-primed control mice.	**(i) Immunohistochemistry assay:** Retinae of infected mice showed microglial relocation in retinal layers and phenotypic changes in morphology of microglial cells.<br>**(ii) Flow cytometry analyses:** A significant increase of cell surface marker expression (i.e.: MHCII, CD45, CD11b) indicating the activation of microglial cells was observed in infected mice compared to control.
