# Supplementary material for: Could Infectious Agents Play a Role in the Onset of Age-related Macular Degeneration? A Scoping Review
Source: Ophthalmol Sci. 2024 Nov 30;5(2):100668. doi: 10.1016/j.xops.2024.100668 (PMC11791433; doi:10.1016/j.xops.2024.100668)
Supplement: Table S3 [file mmc4.pdf]

Table S3: Studies on viruses and other biomarkers included in the review - human studies then experimental studies

**First author**<br>**Year of publication**	**Jabs et al.**<br>**2015**	**Jabs et al.**<br>**2017**	**Ho et al.**<br>**2019**
**Title**	*"Prevalence of intermediate-stage age-related macular degeneration in patients with acquired immunodeficiency syndrome"*	*"Incidence of Intermediate-stage Age-related Macular Degeneration in Patients With Acquired Immunodeficiency Syndrome"*	Letter *"Increased risk of neovascular age-related macular degeneration in patients with herpes zoster ophthalmicus: a retrospective cohort study"*
**Journal**	American Journal of Ophthalmology	American Journal of Ophthalmology	Acta Ophtalmologica
**Infectious agent investigated**	> HIV<br>> Co-infection HCV - HIV	> HIV<br>> Co-infection HCV - HIV	> Varicella Zoster virus
**Original data source**<br>Name<br>+ (if available location, type, baseline or period)	Longitudinal Study of the Ocular Complications of AIDS<br>+ comparison to the Beaver Dam Offspring Study (HIV-uninfected subjects)<br>> Country: USA<br>> Type of data: Prospective cohort study of patients with AIDS<br>> Baseline: between 1998 and 2011	Longitudinal Study of the Ocular Complications of AIDS<br>+ comparison to the Multi-Ethnic Study of Atherosclerosis (HIV-uninfected subjects)<br>> Country: USA<br>> Type of data: Prospective cohort study of patients with AIDS<br>> Baseline: between 1998 and 2011	Longitudinal Health Insurance Database 2005<br>> Country: Taiwan<br>> Type: Data on registration files and medical claims of a representative subsample of insured subjects within the National Health Insurance Research Database<br>> Baseline: no details
**Design**<br>Design<br>Participants (number + main inclusion and exclusion criteria)	> Design: cross-sectional<br>> Participants:<br>*1,825 participants with AIDS in the LSOCA study<br>* Published results of 2810 participants in the Beaver Dam Offspring Study<br>> Exclusion criteria: ocular opportunistic infections, no retinal photograph	> Design: prospective cohort<br>> Participants:<br>* 730 and 379 participants with AIDS with photographs at the 5-year or 10-year follow-up in the LSOCA study, respectively<br>* Published results from 3,685 participants in the Multi-Ethnic Study of Atherosclerosis<br>> Exclusion criteria: ocular opportunistic infections, prevalent AMD, no follow-up	> Design: Propensity score-matched cohort study, 3 year follow-up from index date (The date a patient received their first HZO diagnosis was identified as the index date.)<br>> Participants:<br>*1,148 participants with Herpes zoster ophtalmicus "during an ambulatory visit between 2001 and 2010"<br>*5,740 propensity score-matched controls (1:5)<br>> Exclusion criteria: age < 40, medical history of dry or neovascular AMD prior to index date
**Assessment of the infection**	> AIDS diagnosed according to the 1993 Centers for Disease Control and Prevention revised criteria<br>> HCV serology at inclusion (19% of seropositive subjects)	> AIDS diagnosed according to the 1993 Centers for Disease Control and Prevention revised criteria<br>> HCV serology	> First-time "principal diagnosis" of Herpes zoster ophtalmicus based on ICD-9 code (ICD-9 053.2)
**Assessment of AMD**	> Diagnoses of intermediate-stage AMD (AREDS simple scale stage 3) were made using fundus photographs.	> Diagnoses of intermediate-stage AMD (AREDS simple scale stage 3) were made using fundus photographs.	> Neovascular AMD based on ICD-9 code (ICD-9 362.52)
**Statistical methods**	> Comparisons between the two cohorts: results were stratified by age and adjusted for age and sex.<br>> Within HIV-infected subjects: Multivariate logistic regression models were adjusted for age, HIV transmission category, HCV infection and smoking.	> Comparisons between the two cohorts: Poisson regression was used to estimate the relative risk of AMD adjusted for race/ethnicity and sex (but not age).<br>> Within HIV-infected subjects: Univariate analyses.	> Cox models<br>> Propensity score included sex, age, monthly income, urbanization level, geographical region, hypertension, coronary heart disease, hyperlipidaemia, tobacco use disorder and diabetes
**Results**	> Compared with the HIV uninfected population of the Beaver Dam Offspring Study:<br>*the crude prevalence of AMD is 3-fold higher in the LSOCA cohort (9.9% vs 3.3%)<br>*the age-adjusted and age- and sex-adjusted prevalences were approximately 4-fold higher in LSOCA (12.1% vs 2.9% and 13.0% vs 3.2%, respectively).<br>> Among HIV infected patients,<br>In univariate analysis : The prevalence of AMD was higher in subjects with either injection drug use (14.4%) or heterosexual contact (12.0%) compared to men who had sex with men (7.7%) or other risk groups (5.6%) (p=0.003). Neither co-infection with HCV nor CD4+ T cell count nor HIV load nor the use of any class of antiretroviral drugs were associated with an increased prevalence of AMD.<br>In multivariate analysis : The prevalence of AMD remained higher in subjects with either injection drug use (aOR=2.4 [1.5-3.9], p=0.01) and heterosexual contact (aOR=1.9 [1.3-2.8], p=0.001) compared to men who had sex with men. Co-infection with HCV was not associated with an increased risk of AMD (aOR= 0.7 [0.4-1.1], p=0.15).	> Compared to HIV-uninfected controls, patients with AIDS had an increased risk of intermediate-stage AMD (RR=1.75 [1.16-2.64], p=0.008 after adjustment for race/ethnicity and sex) even though controls were much older than LSOCA participants (61 ± 9 vs 44 ± 8 years).<br><br>> Among HIV-infected participants, neither co-infection with HCV nor count of CD4+ T cells nor HIV load nor HIV transmission type nor HIV treatment were associated with an increased incidence of AMD (univariate analyses).	> Incidence rate of neovascular AMD: 2.31 per 1000 person-years with 6.82 for HZO patients and 1.44 for controls.<br><br>> Risk of neovascular AMD: aHR=4.62 [2.59-8.24], p<0.001 for HZO participants compared to controls

**First author**<br>**Year of publication**	**Miller et al.**<br>**2004**	**Kahn et al.**<br>**1977**	**Roh et al.**<br>**2008**
**Title**	*"The association of prior cytomegalovirus infection with neovascular age-related macular degeneration"*	*"The Framingham Eye Study. Association of ophtalmic pathology with single variables previously measured in the Framingham Heart Study"*	*"Estimated Prevalence and Risk Factor for Age-related Maculopathy"*
**Journal**	American Journal of Ophthalmology	American Journal of Epidemiology	Yonsei Med J
**Infectious agent investigated**	> Cytomegalovirus<br>> C. pneumoniae<br>> H. pylori	> History of lung infections	> Hepatitis B Virus<br>> Hepatitis C Virus
**Original data source**<br>Name<br>+ (if available location, type, baseline or period)	> Country: USA<br>> Type: Case control study including patients examined at the Bascom Palmer Eye Institute or the Bascom Palmer Palm Beach Clinic<br>> Baseline: October 2001 to December 2002	The Framingham Heart and Eye Studies<br>> Country: USA<br>> Type: Prospective cohort<br>> Baseline: 1948 for the Heart Study and 1973 for the Eye Study	The Yonsei Eye Study<br>> Country: South Korea (Seoul)<br>> Type: Cross-sectional survey<br>> Baseline: 2006
**Design**<br>Design<br>Participants (number + main inclusion and exclusion criteria)	> Design : Case-control study<br>> Participants:<br>*47 neovascular AMD<br>*36 dry AMD<br>*67 controls<br>>Exclusion criteria: age <50 or >90, HIV infection, malignancy, recent acute illness requiring hospitalization within 6 months, immunosuppressive condition	> Design: Prospective cohort<br>> Participants: no details<br>NB : Analyzes concerning AMD and "a history of pulmonary infection at follow-up V (1956-1960)" presented on 972 subjects<br>> Exclusion criteria: no details	> Design: Cross-sectional<br>> Participants: 9,530 participants<br>> Inclusion criteria: age >= 40, full clinical examinations
**Assessment of the infection**	> Analysis of cytomegalovirus, *C. pneumoniae*, and *H. pylori* IgG antibody titers by ELISA in the serum. Participants were not classified as positive or negative but rather in low, medium and high tertiles of IgG (thresholds defined in the control group)	> No details provided.<br>Exposure variables collected during the Framingham Heart Study between 1948-1964 (monitored every two years).	> Serum hepatitis B surface antigen status (HBsAg) (reflecting present HBV infection)<br>> Serum hepatitis B core antibody level (HBcAb) (reflecting present HBV infection or past HBV infections)<br>> Serum hepatitis B surface antibody level (HBsAb) (reflecting vaccination against HBV or past HBV infection)<br>> Hepatitis C antibody level (HCVAb) (reflecting present or past HCV infection)<br>NB: In the study, 4.6% of HBsAg carriers (n=428 including 18 AMD) and 0.8% of HCVAb carriers (n=79 including 1 AMD)
**Assessment of AMD**	> Diagnoses of dry and wet AMD (using the International Classification for Age-related Macular Degeneration) were made using fundus photographs and fluorescein angiography if appropriate.	> Diagnoses of senile macular degeneration (no details) were made by ophthalmological examination during the Framingham Eye Study in 1973-1975	> Diagnoses of AMD (using the Wisconsin Age-Related Maculopathy Grading system) were made using fundus photographs.<br>NB : Prevalence of AMD : 2.46% (2.3% for early AMD and 0.2% for late AMD)
**Statistical methods**	> Student's test for independent samples<br>> NB : Results on the total pathogen exposure not shown here	> Univariate analyzes on data stratified by sex and age categories	> Logistic regression models<br>> Full adjustement on age, sex, monthly income, education, BMI, hypertension, current smoking, CRP, triglycerides, HDL<br>> Reduced adjustement excluding BMI, CRP, triglycerides and HDL
**Results**	> Subjects with wet AMD had higher levels of anti-CMV IgG than both controls (p=0.02) and subjects with dry AMD (p=0.06). There was no difference between subjects with dry AMD and controls (p=0.83).<br>> The proportion of subjects with high titers of anti-CMV IgG was higher among subjects with wet AMD compared to both dry AMD (55% vs 39%, OR=2.23 [0.77-6.44]) and controls (55% vs 34%, OR=2.49 [0.98-6.33]). There was no significant difference in the distribution of CMV IgG titers in dry AMD versus controls (OR=1.12 [0.42-2.94]).	> History of lung Infection on exam V (1956-1960) was associated with senile macular degeneration in men aged 52 - 64 and in women aged 65-74 during the Framingham Eye Study in 1973-1975.	> In age-adjusted models, association with AMD:<br>*HBsAg aOR=2.65 [1.59-4.43] p<0.001<br>*HBcAb aOR=1.48 [1.09-1.99] p=0.011<br>*HBsAb aOR=0.92 [0.69-1.22] p=0.55<br>*HCVAb aOR=0.19 [0.03-1.41] p=0.10<br>> In multivariate models, association with AMD:<br>* HBsAg aOR=2.56 [1.48 - 4.42] with full adjustment, p not reported<br>* HBsAg aOR=2.74 [1.59-4.71] with reduced adjustment, p not reported

First author<br>Year of publication	Park et al.<br>2014	Chou et al.<br>2018
**Title**	*"Age-related macular degeneration: Prevalence and Risk Factors from Korean National Health and Nutrition Examination Survey, 2008 through 2011"*	*"HBV infection increases the risk of macular degeneration: the roles of HBx-mediated sensitization of retinal pigment epithelial cells to UV and blue light irradiation"*
**Journal**	Ophthalmology	Journal of Translational Medicine
**Infectious agent**	> Hepatitis B virus	> Hepatitis B Virus
**Original data source**<br>Name<br>+ (if available location, type, baseline or period)	The Korean National Health and Nutrition Examination Survey<br>> Country: South Korea<br>> Type: Annual cross-sectional surveys<br>> Period: from 2008 through 2011	
**Design**<br>Design<br>Participants (number + main inclusion and exclusion criteria)	> Design: Cross-sectional<br>> Participants: 14,352 participants<br>> Inclusion criteria: age >= 40, participation in the 4-year study period, gradable fundus photograph of at least one eye	> Design: Frequency-matched (1:4) cohort study, follow-up until diagnosis of macular degeneration (MD) (not restricted to AMD), withdraw of database or the end of 2011<br>> Participants:<br>*39,796 participants who received a first-time diagnosis of HBV infection between January 1, 2000 and December 31, 2011<br>*159,184 controls matched by age and year of index date<br>> Exclusion criteria : age<20, HCV infection, MD before the index date (date of first diagnosis of chronic HBV infection).
**Assessment of the infection**	> Presence of hepatitis B surface antigen (HBsAg) in the blood (reflecting present HBV infection)<br><br>NB: In the study, 3.9% of HBsAg carriers	> First-time diagnosis of (acute or chronic) HBV infection using the following ICD-9 codes:<br>070.20 "Viral hepatitis B with hepatic coma, acute or unspecified, without mention of hepatitis delta"<br>070.22 "Viral hepatitis B with hepatic coma, chronic, without mention of hepatitis delta"<br>070.30 "Viral hepatitis B without mention of hepatic coma, acute or unspecified, without mention of hepatitis delta"<br>070.32 "Viral hepatitis B without mention of hepatic coma, chronic, without mention of hepatitis delta"<br>V02.61 "Hepatitis B carrier"
**Assessment of AMD**	> Diagnoses of early and late AMD (using the grading protocol of the International Age-Related Maculopathy Epidemiological Study Group) were made using fundus photographs.<br>NB : Prevalence of AMD: 6.62% (6.02% for early AMD and 0.60% for late AMD)	> MD based on ICD-9 code (362.5)
**Statistical methods**	> Logistic regression models<br>> Step 1: models were adjusted on age group, sex and smoking status<br>> Step 2: models were adjusted on age group, sex and smoking + variables with a p-value <0.1 in step 1<br>For early AMD:<br>Model 1: age group, sex, smoking status, household income, education, occupation, diabetes mellitus, dyslipidemia, BMI, anemia, creatinine and HBsAg<br>Model 2: age group, sex, smoking status, household income, education, occupation, diabetes mellitus, dyslipidemia, waist circumference, anemia, creatinine and HBsAg<br>For late AMD: age group, sex, smoking status, and BMI (not HBsAg)	> Cox model<br>> Adjustment for age, sex, and comorbidities (including hypertension, hyperlipidemia,diabetes, asthma, cirrhosis, anxiety and coronary artery disease)<br>> Stratified analyses on age group, sex and presence of at least one comorbidity
**Results**	> Step 1: models adjusted for age group, sex and smoking status<br>Early AMD: HBsAg aOR=1.91 [1.33-2.75] p =0.001<br>Late AMD: HBsAg aOR=1.49 [0.51-4.32] p=0.467<br><br>> Step 2: fully adjusted models only for early AMD<br>HBsAg aOR=1.98 [1.38-2.85] p <0.001 (model 1)<br>HBsAg aOR=1.96 [1.36-2.83] p <0.001 (model 2)	> Incidence of MD in HBV-infected patients 1.90 per 1000 person-years *versus* 1.47 in controls<br><br>> In multivariate models, association with MD<br>*aHR 1.31 [1.17–1.46] p<0.001<br>*Among patients aged ≥ 50 years: aHR 1.24 [1.10–1.40] p<0.001 while NS among patients aged ≤ 35 and in those aged 35–49 years.<br>*The associations remained statistically significant after stratification on sex and the presence of comorbidities.

**First author**<br>**Year of publication**	**Wu et al.**<br>**2019**	**Yeh et al.**<br>**2021**
**Title**	*"Association of chronic hepatitis B virus infection with age-related macular degeneration"*	*"Increased Risk of Age-Related Macular Degeneration with Chronic Hepatitis C Virus Infection: A Nationwide Population-Based Propensity Score-Matched Cohort Study in Taiwan"*
**Journal**	Acta Ophtalmologica	Viruses
**Infectious agent**	> Hepatitis B Virus	> Hepatitis C virus
**Original data source**<br>Name	Longitudinal Health Insurance Database 2000<br>> Country: Taiwan<br>> Type: Registration files and medical claims (inpatient and oupatient files, drug use…) of a representative sample of 1 million insured subjects within the National Health Insurance Research Database<br>> Baseline: 2000	
**Design**<br>Design<br>Participants (number + main inclusion and exclusion criteria)	> Design: Frequency-matched (1:4) cohort study, follow-up until diagnosis of AMD, withdraw of database or the end of 2013<br>> Participants:<br>*17,796 participants who received a first-time diagnosis of chronic HBV infection between January 1, 2000 and December 31, 2012<br>*71,184 controls matched by age, sex and year of index date<br>> Exclusion criteria : age <40, chronic hepatitis C virus infection, HIV infection, progressive high myopia or a history of any type of AMD prior to the index date (date of first diagnosis of chronic HBV infection).	> Design : Propensity score-matched cohort study, follow-up until diagnosis of AMD, withdraw of database or the end of 2013<br>> Participants:<br>*13,300 participants who received a first-time diagnosis of chronic HCV infection between January 1, 2000, and December 31, 2012<br>*26,600 propensity score-matched participants without HCV<br>+ Among the "HCV cohort" : 1,973 participants with HCV who completed pegylated interferon and ribavirin treatment were matched by propensity-score to 3,946 untreated patients with HCV<br>> Exclusion criteria: age <18 years, HBV infection, HIV infection, history of progressive high myopia, AMD before index date (date of first diagnosis of chronic HBV infection).
**Assessment of the infection**	> First-time diagnosis of chronic HBV infection using the following ICD-9 codes:<br>070.2 "Viral hepatitis B with hepatic coma"<br>070.3 "Viral hepatitis B without mention of hepatic coma"<br>V02.61 "Hepatitis B carrier"<br>> Excluding participants with "only one diagnosis of acute or unspecified HBV:<br>070.20 "Viral hepatitis B with hepatic coma, acute or unspecified, without mention of hepatitis delta"<br>070.21 "Viral hepatitis B with hepatic coma, acute or unspecified, with hepatitis delta"<br>070.30 ""Viral hepatitis B without mention of hepatic coma, acute or unspecified, without mention of hepatitis delta"<br>070.31 "Viral hepatitis B without mention of hepatic coma, acute or unspecified, with hepatitis delta"<br>> NB : Patients with a second diagnosis of HBV infection 6 months after a diagnosis of acute or unspecified HBV were considered as having chronic HBV.	> First-time diagnosis of chronic HCV infection using ICD-9 codes<br>070.41 "Acute hepatitis C with hepatic coma"<br>070.44 "Chronic hepatitis C with hepatic coma"<br>070.51 "Acute hepatitis C without mention of hepatic coma"<br>070.54 "Chronic hepatitis C without mention of hepatic coma"<br>070.70 "Unspecified viral hepatitis C without hepatic coma"<br>070.71 "Unspecified viral hepatitis C with hepatic coma"<br>V02.62 "Hepatitis C carrier"<br>> Excluding participants with "only one diagnosis of acute or unspecified HCV infection" (070.41, 070.51, 070.70, 070.71).<br>"Patients who received a second diagnosis of HCV infection within 6 months after being diagnosed as having acute or unspecified HCV infection were considered as having chronic HCV."
**Assessment of AMD**	> AMD based on ICD-9 codes (362.42, 362.43, 362.52, 362.53, 362.50 and 362.51)<br>> Analyzes subdividing :<br>*exudative and nonexudative AMD<br>*types of evolution during follow-up: exudative only, exudative first followed by nonexudative, and nonexudative first followed by exudative	> AMD based on ICD-9 codes (362.42, 362.43, 362.52, 362.53, 362.50 and 362.51)<br>> Analyzes on exudative and nonexudative types
**Statistical methods**	> Cox model<br>> Adjustment for age group, sex, socioeconomic factors (including occupation category, urbanization level, region of residence, and monthly income), comorbidities (including hypertension, diabetes mellitus, diabetic retinopathy, hyperlipidaemia, chronic obstructive pulmonary disease, coronary heart disease, chronic renal disease, stroke, cataract), and the use of statin, aspirin, and other nonsteroidal anti-inflammatory drugs.<br>> Stratified analyses on age group and sex	> Cox model<br>> Propensity score for the HCV vs non-HCV matching included age, sex, occupation, urbanization, monthly income, index year, myocardial infarction, cerebrovascular disease, diabetes mellitus, renal disease, hypertension, hyperlipidemia, liver cirrhosis, anemia, statin use<br>> Propensity score for the treated vs untreated HCV matching included age, sex, monthly income, index year, myocardial infarction, cerebrovascular disease, renal disease, cataract, liver cirrhosis, anemia, statin use<br>> Stratified analyzes on age group and sex.<br>> Sensitivity analysis excluding AMD cases within the 1st year after index date
**Results**	> Incidence of any type of AMD: 3.88 per 1000 person-years (1.61 per 1000 person-years in the non-HBV cohort 2.27 per 1000 person-years in the HBV cohort).<br>> In multivariate models, associations with AMD:<br>For any type: aHR 1.41 [1.23–1.63] p<0.001<br>For exudative (E) : aHR 1.43 [1.08–1.89] p=0.012<br>For non-exudative (NE): aHR 1.41 [1.19–1.65] p<0.001<br>For E only: aHR 1.25 [0.80–1.97] p=0.329<br>For progression from E to NE: aHR 1.45 [0.91–2.33] p=0.120<br>For progression from NE to E: aHR 1.74 [1.01–2.99] p=0.048<br>> The median numbers of days for the progression from NE to E AMD were 656 (31–3248) for the non-HBV cohort and 605 (31–3514) for the HBV cohort.<br>> Depending on the type of AMD studied, certain associations were no longer statistically significant after stratification on age and sex.	> "The median follow-up duration from the index date to the diagnosis of any type of AMD was 5.74 and 5.83, years, respectively, in the non-HCV and HCV cohorts, respectively."<br>> "The incidence of any type of AMD was 11.89 per 1000 PYs, which was equal to the combined incidence for both the non-HCV (5.32 per 1000 PYs) and HCV (6.57 per 1000 PYs) cohorts."<br>> HCV-infected vs HCV-uninfected cohorts (aHR with 95% CI)<br>For any type of AMD: 1.22 [1.09–1.35], p=0.0004<br>For exudative AMD: 1.17 [0.84-1.61], p=0.354<br>For nonexudative AMD: 1.22 [1.09–1.37], p=0.0006<br>> HCV-treated vs HCV-untreated cohorts (aHR with 95% CI)<br>For any type of AMD: 1.07 [0.81–1.43], p=0.240<br>For exudative AMD: 1.36 [0.58–3.22], p=0.497<br>For nonexudative AMD: 1.04 [0.77–1.41], p=0.781<br>> Other results available on age and sex specific risks. Sensitivity analyses: similar results

First author<br>Year of publication	Chou et al.<br>2018	Cousins et al.<br>2012	
**Title**	*"HBV infection increases the risk of macular degeneration: the roles of HBx-mediated sensitization of retinal pigment epithelial cells to UV and blue light irradiation"*	*"Macrophage activation associated with chronic murine cytomegalovirus infection results in more severe experimental choroidal neovascularization"*	
**Journal**	Journal of Translational Medicine	PLoS pathogens	
**Type**	In vitro	Animal	In vitro
**Infectious agents**	Hepatitis B virus	Cytomegalovirus	Cytomegalovirus
**Cell line or animal models<br>Exposition**	> Cell line : ARPE19, a human retinal pigment epithelial cell line<br>> Exposition : HBx-transfected cells (overexpressing HBx, the pathogenic X protein of HBV) vs mock-transfected cells	> Animal model: C57BL/6 mice<br>> Exposition:<br>Firstly, mice were peritoneally infected with murine CMV (MCMV). Controls were infected with UV-inactivated MCMV (or mock-infected for the experiments involving ganciclovir).<br>Secondly, mice were subjected to laser treatment of the eye to induce choroidal neovascularization either 6 days (acute infection), 6 weeks or 12 weeks post-infection (chronic infections)	> Cell line: mouse macrophage cell line of C57BL/6 origin<br>> Exposition: direct MCMV infection (or UV-inactivated MCMV or mock-infected or treated with LPS)
**Main outcomes and results**	**(i) Comparison of cell viability and clonogenic survival (measured using MTT assay and colony formation asay) after exposition to either UV or blue light irradiation:**<br>Compared to mock-transfected cells, HBx-transfected cells (overexpressing HBx) had significantly reduced cell viability and clonogenic survival upon UV and blue light irradiation.<br><br>**(ii) Comparison of the gene expression profiles after UV irradiation + enrichment analysis to functionally classify differentialy expressed genes:**<br>Compared to mock-transfected cells, HBx-transfected cells showed alterations of gene expression profiles in signaling pathways including:<br>*prostaglandin metabolism, cytokine–cytokine receptor interaction, extracellular matrix–receptor interaction, steroidogenesis, and the PI3K-Akt signaling pathway which were particularly altered before UV irradiation<br>* base excision repair, nucleotide excision repair, mismatch repair, and homologous recombination, DNA replication, cell cycle regulation, and circadian rhythms which were particularly altered after UV irradiation<br>*the p53, TNF, and cancer-related pathways were altered both pre- and post-UV irradiation.<br>The results suggest that the negative impact of HBx on RPE cell viability may implicate down-regulation of various DNA repair pathways.	**(i) Severity of CNV 4 weeks after its induction by laser treatment**: Infected mice presented larger CNV lesions and their size were positively correlated with duration of infection before laser treatment.<br><br>**(ii) Detection of MCMV DNA using PCR in choroidal tissues (as well as in spleen, lung and salivary gland tissues, splenic macrophages, cells from bone marrow):** MCMV DNA could not be found in choroidal tissues at time of chronic MCMV infection (i.e. when CNV lesions were the most severe). Conversely, MCMV DNA was found in the other tissues studied including splenic macrophages.<br><br>**(iii) Real time RT-PCR assay performed on splenic macrophages of chronically infected mice to detect and quantify transcripts of pro-inflammatory and pro-angiogenic cytokines:** Higher levels of transcripts of proinflammatory and pro-angiogenic factors (such as TNFa as well as MMP-9, COX2 and VEGF which are proteins involved in angiogenesis) were found in splenic macrophages of chronically infected animals compared to controls.<br>NB: Splenic macrophages were used because sufficient numbers of macrophages could not be collected from eyes.<br><br>**(iv) Impact of ganciclovir treatment, an antiviral inhibiting MCMV replication:** Ganciclovir significantly inhibited the production of VEGF and TNFa mRNA, suggesting an active viral replication in these processes.	**(i) Detection of TNF-a and VEGF mRNA by quantitative RT-PCR assay and VEGF protein by ELISA and immunostaining 24 and 48h post-infection:** Direct MCMV infection upregulated the production of VEGF mRNA and VEGF protein but failed to duplicate an increase in TNFa mRNA. Compared to mock-infected cells, MCMV-infected macrophages also had increased levels of IL-10 and IL-1RA mRNA, equivalent levels of IL-23 mRNA production, and no detectable IL-21 mRNA production, which is consistent with a M2 phenotype of macrophage activation, a proangiogenic phenotype.<br><br>**(ii) Impact of ganciclovir treatment:** VEGF production was sensitive to ganciclovir (an antiviral that inhibits MCMV replication).

First author<br>Year of publication	Zinkernagel et al.<br>2013	Xu et al.<br>2020	
**Title**	*"Interferon γ-dependent migration of microglial cells in the retina after systemic cytomegalovirus infection"*	*"Ocular cytomegalovirus latency exacerbates the development of choroidal neovascularization"*	
**Journal**	The American Journal of Pathology	The Journal of Pathology	
**Type**	Animal	Animal	Human cadavers
**Infectious agents**	Cytomegalovirus	Cytomegalovirus	Cytomegalovirus
**Cell line or animal models**<br>**Exposition**	> Animal models:<br>(i) BALB/c or congenic CT6 BALB/c mice<br>(ii) Knock-out mice for IFNg or MyD88<br>(iii) Generation of bone marrow chimera models<br>> Exposition: intraperitoneal inoculation of murine CMV (or CpG alone, a motif shown to activate murine immune cells)	> Animal models: wild-type(WT) mice and vascular endothelial growth factor (VEGF)-overexpressing VEGF-A^{hyper} mice<br><br>> Exposition: intraperitoneal inoculation of murine CMV (or culture medium as control) at <3days after birth	> Human samples: 24 pairs of fresh eyes from human cadavers (with no history of AMD mentioned)
**Main outcomes and results**	**(i) Quantification and characterization of microglial cells in the retina +/- the iris using immunofluorescence, epifluorescence or confocal microscopy and flow cytometry**:<br>> Systemic MCMV infection was associated with an increased number of microglial cells in the subretinal space and with some incipient damage to the photoreceptor outer segment. Both morphological changes and flow cytometry data suggested that systemic MCMV infection also induced the activation of these microglial cells.<br>> An increase in the number of subretinal microglial cells was also highlighted in the absence of viral infection by administration of CpG alone (which can activate immune cells).<br>> Systemic MCMV infection was also associated with an increased number of iris macrophages.<br><br>**(ii) Search for MCMV genome using real time PCR in the retina:** No MCMV DNA was detected in the retina.<br><br>**(iii) Exploration of the involvement of IFN-g and MyD88 (implicated in the Toll-like receptor signaling pathway) using knockout mice for IFN-g and MyD88:** Experiments suggested that the migration of microglial cell into the subretinal space is dependent on IFN-g and MyD88.<br><br>**(iv) Exploration of the origin of microglial cells using bone marrow chimera experiments:** Following MCVM infection, accumulation of microglial cells into the subretinal space was due to migration of resident retinal microglia from the inner neural retina (and not from the circulating monocyte pool).	**(i) Determination if neonatal systemic MCMV infection leads to virus dissemination and latency in the eye**: At 14 days post-infection, replicating MCMV and MCMV DNA were found in the majority of the eyes in both VEGF-A^{hyper} and WT infected mice, mainly in the choroid and sclera. At 6 months post-infection, no replicating MCMV was recovered but MCMV DNA was found in all eyes of both groups of mice. Nevertheless, MCMV immediate early gene (IE1 but not IE3 or late gene gB) was detected only in infected VEGF-A^{hyper} mice (and not in WT). NB: Results also available for some other tissues.<br><br>**(ii) Search for the presence of choroidal neovascularization by spectral-domain optical coherence tomography (SDOCT) and fluorescein angiography**: While no CNV lesions were observed in WT mice (infected or not) at 6 months post-infection, infected VEGF-A^{hyper} mice showed significantly more frequent and more severe CNV lesions compared to uninfected VEGF-A^{hyper} mice. In contrast, there was no significant difference regarding photoreceptor degeneration.<br><br>**(iii) Search for other pathological changes by electron microscopy**: At 6 months post-infection, changes in the morphology of photoreceptors were seen in areas affected by CNV lesions (shortening and loss of outer segments) and pigmented RPE-like cells were shown to profilerate and/or migrate along new vessels into the inner retina.<br><br>**(iv) Determination of VEGF-A levels in eyes by ELISA:** At 6 months post-infection, infected VEGFA^{hyper} mice had significantly higher levels of VEGF-A in the eyes than uninfected VEGFA^{hyper} mice. Conversely, no differences was found between infected and uninfected WT mice.<br><br>**(v) Determination of a potential recruitment of macrophages to CNV lesions by immunofluorescence staining (VEGF and F4/80, a macrophage marker):** While macrophages were rarely observed in eyes of WT mice, similar numbers of macrophages were observed in CNV lesions of infected and uninfected VEGF-A^{hyper} mice. Notably, even though some of these macrophages were VEGF-positive, they did not represent the majority of VEGF-positive cells.<br><br>**(vi) Determination of levels of inflammatory/angiogenic factors by western blot and ELISA:** At 6 months post-infection, latent ocular MCMV infection was associated with an increased in the levels of ocular RIP3, CCL5, TGF-β1 and IL-6.	**(i) Search for presence of HCMV DNA using droplet digital PCR assay:** CMV DNA was detected in posterior eye cups (choroid/RPE) of 4 individuals out of 24 (17%), leading the authors to suggest that the choroid/RPE is a fairly common site of latency for CMV. Very low DNA copy numbers were detected in the neural retina of a fifth individual (4,2%). Conversely, CMV DNA was not found in any of the anterior segment samples.

First author<br>Year of publication	Xu et al.<br>2021	Zhang et al.<br>2023
**Title**	*"Retinal and Choroidal Pathologies in Aged BALB/c Mice Following Systemic Neonatal Murine Cytomegalovirus Infection"*	*"Transcriptome Analysis of Retinal and Choroidal Pathologies in Aged BALB/c Mice Following Systemic Neonatal Murine Cytomegalovirus Infection"*
**Journal**	The American Journal of Pathology	International journal of molecular science
**Type**	Animal	Animal
**Infectious agents**	Cytomegalovirus	Cytomegalovirus
**Cell line or animal models**<br>**Exposition**	> Animal models: BALB/c mice<br>> Exposition: intraperitoneal inoculation of murine CMV (or culture medium as control) at <3days after birth	> Animal models : BALB/c mice<br>> Exposition : intraperitoneal inoculation of murine CMV (or culture medium as control) at <3days after birth.
**Main outcomes and results**	**(i) Search for replicating virus (by plaque assay), viral DNA (by PCR) and MCMV early antigen expression (by immunofluorescence staining or immunogold electron microscopy):** At 14 days post-infection, replicating virus was detected in eyes of all mice. MCMV early antigen was present in some vascular endothelial cells and pericytes in the choriocapillaris as well as in sporadic RPE cells. At 3 months post-infection, while no replicating virus was detected, MCMV DNA was detected in the sclera and choroid of all eyes and some RPE cells. In latently infected mice which were immunosuppressed using methylprednisolone plus anti-T-cell antibodies, replicating virus was found in 6 of 8 eyes and MCMV DNA was detected in the choroid (6/6 mice) and in RPE cells (3/6 mice).<br>NB: Results for other tissues and results of electron microscopy assessing pathological changes associated with acute infection were not reported here.<br><br>**(ii) Search for pathologic changes associated with latent ocular infection (using spectral domain optical coherence tomography and electron microscopy):** In latently infected mice, mean retinal thickness was significantly reduced compared with age-matched control. While no other pathologic change was found at 4 or 8 months post-infection, severe photoreceptor degeneration was observed at 18 months post-infection in 21 of 40 eyes as well as CNV-like lesions (in 6 eyes out of 21) and retinal detachment (in 4 eyes out of 21). Using electron microscopy, at 18 months post-infection, several changes were described including loss of choroidal capillaries, infiltrating cells in the subretina, deposits at the basal and apical aspects of the RPE, degeneration of choroidal endothelia, RPE, and photoreceptors, presence of CNV-like lesions and severe retinal degeneration.<br><br>**(iii) Search for expression of MCMV genes (using real-time RT-PCR):** At 8 and 18 months post-infection, expressions of some MCMV genes (IE1 and IE3 as well as genes with an antiapoptotic or an immunomodulating role) were detected in eyes of infected mice.<br><br>**(iv) Search for expression of genes related to inflammatory/angiogenetic factors (using real-time RT-PCR and ELISA)**: In latently infected mice, levels of CCL5 transcripts (at 8 and 18 months post-infection) as well as increased levels of IL6 and CCL7 (at 18 months post-infection only) were increased compared to controls. Moreover, higher protein levels were also reported for CCL5 (at 8 and 18 months post-infection) and IL6 (at 18 months post-infection).<br><br>**(v) Search for infiltration of mononuclear macrophages into the eye by staining (Iba1 and F4/80)**: From the 4th month post-infection, the presence of F4/80-positive mononuclear phagocytes was highlighted in the subretinal space and outer nuclear layer.	**(i) Determination of molecular genetic changes and pathways affected by ocular MCMV latency (using RNA sequencing analysis):** 321 differentially expressed genes (208 downregulated and 113 upregulated) were identified in latently infected eyes compared to controls. QIAGEN Ingenuity Pathway Analysis highlighted 17 affected canonical pathways including 10 implicated in neuroretinal signaling and 7 in upregulated immune/inflammatory responses. Among the differentially expressed genes (DEGs), some were implicated in retinal and epithelial cell death pathways (either by apoptosis or necrosis) as well as in migration, infiltration, and activation of multiple immune cell types.<br>Comparing infected eyes with and without severe retinal degeneration, 48 differentially expressed genes were identified of which some were involved in pathways related to neuroretinal signaling, cell death, and retinal degeneration (and not in immune response pathways).<br>NB: The contribution of both aptotosis and necroptosis were confirmed by TUNEL assays and Western blots targeting cleaved caspase 3, RIP3, MLKL and rhodopsin. The accumulation of macrophages/microglia in the subretinal space and outer nuclear layer in infected mice was confirmed by immunstaining.<br><br>**(ii) Analysis of upstream regulators of the differentially expressed genes:** 36 upstream regulators were identified including cytokines (IL1, IFN, IL17A, TNF, etc.), transcription factors involved in innate immunity/inflammation (STAT1, STAT3) and several growth factors (VEGF, TGF).

First author<br>Year of publication	Brosig et al.<br>2015	Hoh Kam et al.<br>2016
**Title**	"*Gene expression regulation in retinal pigment epithelial cells induced by viral RNA and viral/bacterial DNA*"	*"Aged complement factor H knockout mice kept in a clean barriered environment have reduced retinal pathology"*
**Journal**	Molecular Vision	Experimental Eye Research
**Type**	In vitro	Animal
**Infectious agents**	Viral RNA and viral/bacterial DNA	Conventional open environment versus barriered pathogen free environment
**Cell line or animal models**<br>**Exposition**	> Cell line: Cultured human RPE cells<br>> Exposition: analogs of viral double-stranded RNA (synthetic poly(I:C)) and viral/bacterial DNA (CpG-ODN)"	> Animal models: Complement factor H knock out (Cfh-/-) mice on a C57Bl/6J background<br>> Exposition: Mice were maintained over 9 months in either a conventional open environment (CE) or a specific pathogen free environment (SPF).
**Main outcomes and results**	**(i) Modifications in gene expression and protein secretion determined with real-time RT–PCR and ELISA**:<br>> Viral RNA induced an upregulation of the expression of TLR genes (TLR3 and, to a lower degree, TLR2 but not TLR9), transcription factor genes (HIF-1α, p65/ NF-κB, and NFAT5), angiogenic growth factor genes (bFGF but not VEGF or HB-EGF), proinflammatory cytokine genes (IL-1β, IL-6, TNFα, monocyte chemoattractant protein-1, and macrophage inflammatory protein-2), complement factor genes (C5, C9, CFB, and CFH genes but not C3). Viral RNA also induced the secretion of bFGF and TNFα.<br>> Viral/bacterial DNA induced the expression of transcription factor genes (p65/ NF-κB and NFAT5) and complement factor genes (C5 and C9) but did not significantly alter the expression of TLR genes, angiogenic growth factor genes or proinflammatory cytokine genes.<br><br>**(ii) Analyses also investigated the impact of viral dsRNA on activation of intracellular signal transduction pathways (not reported)**.	**(i) Number of macrophages in the outer retina measured by immunostaining (using Iba-1, a marker of macrophage/microglia):** Compared to SPF mice, the number of outer retinal macrophages was significantly higher in CE mice.<br><br>**(ii) Levels of TNF-α and IL-1β (two pro-inflammatory cytokines which can be produced by activated macrophages) in the outer retina measured by immunostaining and western blot**: Compared to SPF mice, the levels of TNF-α and IL-1β in the outer retina were significantly higher in CE mice.<br><br>**(iii) Levels of GFAP and vimentin (two stress markers) in the inner retina measured by immunostaining and wester blot**: Compared to SPF mice, the levels of GFAP and vimentin in the inner retina were significantly higher in CE mice.<br><br>**(iv) Number of photoreceptors across the outer nuclear layer:** Compared to SPF mice, the number of photoreceptors in the outer nuclear layer was significantly reduced in CE mice.<br><br>**(v) Level of amyloid beta deposition on Bruch's membrane measured by immunostaining:** There was no difference regarding amyloid beta deposition on Bruch's membrane between the two groups.
